# Control over the relative reactivities of monomers in RAFT copolymerization of styrene and acrylic acid†

**DOI:** 10.1039/c8ra00048d

**Published:** 2018-04-18

**Authors:** E. V. Chernikova, S. D. Zaitsev, A. V. Plutalova, K. O. Mineeva, O. S. Zotova, D. V. Vishnevetsky

**Affiliations:** Polymer Department, Faculty of Chemistry, Lomonosov Moscow State University Lenin Hills, 1, bld.3 Moscow 119991 Russian Federation chernikova_elena@mail.ru; Nizhni Novgorod State University pr. Gagarina, 23 Nizhni Novgorod 603950 Russian Federation szay@inbox.ru; Tver State University Zhelyabova st., 33 Tver 170100 Russian Federation

## Abstract

The relative monomer reactivities in the reversible addition–fragmentation chain transfer (RAFT) radical copolymerization of styrene (S) and acrylic acid (AA) in a solution of the polar solvent *N*,*N*-dimethylformamide are found to be dependent on the chemical nature of the RAFT agent. Polymeric RAFT agents based on polyacrylic acid enhance the difference in monomer reactivities (dithiobenzoate – *r*_AA_ = 0.09 ± 0.02, *r*_S_ = 3.5 ± 1.2, trithiocarbonate – *r*_AA_ = 0.08 ± 0.04, *r*_S_ = 3.03 ± 1.78) compared to low molecular weight RAFT agents (dibenzyl dithiobenzoate – *r*_AA_ = 0.14 ± 0.01, *r*_S_ = 1.00 ± 0.01, dibenzyl trithiocarbonate – *r*_AA_ = 0.08 ± 0.01, *r*_S_ = 0.85 ± 0.03). The opposite effect on the relative reactivity of acrylic acid is observed when polymeric RAFT agents based on polystyrene are used (dithiobenzoate – *r*_AA_ = 3.3 ± 0.4, *r*_S_ = 0.72 ± 0.05, trithiocarbonate – *r*_AA_ = 0.11 ± 0.01, *r*_S_ = 0.54 ± 0.03). In all the investigated systems the copolymers formed are characterized by narrow MWD due to the high efficiency of the chosen RAFT agents.

## Abstract 2

The tuning of the relative monomer reactivities of styrene and acrylic acid in a solution of *N*,*N*-dimethylformamide by using polymeric RAFT agents.

## Graphical abstract

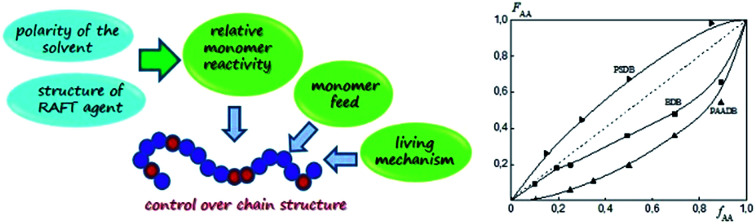

## Introduction

The recent progress in reversible-deactivation radical polymerization (RDRP) is based on knowledge of its main features and mechanisms.^1–3^ As a result, the different types of RDRP techniques are applied successfully for the synthesis of macromolecules with complex architectures, in particular, block- and graft-copolymers of various topologies.^4–9^ In the meantime, the binary copolymerization which proceeds *via* the reversible-deactivation radical mechanism remains less studied. However, controlling the copolymer structure *via* control over the relative reactivities of monomers in RDRP may become a powerful instrument in the fine-tuning of polymer properties.

In principal, the control of propagation in the conventional radical copolymerization of polar and non-polar monomers due to solvent or additive was discovered in the 1970–1980s.^10–16^ This may occur as a result of the formation of the complexes between the monomer and/or propagating radical and the solvent or additive, the solvation of the transition state and the preferential solvation of the reactant. It is clear that these mechanisms can be realized in RDRP. Multiple examples of solvent effects which influence the relative monomer reactivities in the radical polymerization of monomers with different polarities are summarized by E. L. Madruga.^17^

With respect to the mechanism of preferential solvation of the reactant proposed and developed by Harwood, Semchikov and Plochocka,^16–20^ it is supposed that the local concentration of the monomer near the active center changes compared to its average concentration in the medium due to preferential monomer sorption. This affects the relative monomer reactivity and leads to a change in copolymer composition.

This solvent effect, or so-called bootstrap effect, can be observed in the case of bulk copolymerization when one of the comonomers is the good solvent for the copolymer formed and another is the bad solvent. The nature of this kind of mixed solvent and its thermodynamic quality will change during monomer conversion, as monomer composition will vary.^21^ More often this situation can be observed in the bulk copolymerization of hydrophobic and hydrophilic monomers, *e.g.* styrene–acrylic acid, styrene–methacrylic acid and styrene–acrylamide. It is supposed that the hydrophilic monomer preferentially sorbs near the active center due to hydrogen bonding, which leads to a change in its local concentration. Obviously this effect manifests more strongly with an increase in chain length. In practice, the copolymer composition depends on the molecular weight in certain copolymerizations. In order to demonstrate this effect the molecular weight should be varied over a wide range by changing the initiator concentration or by adding a transfer agent.^19,20,22^

The preferential sorption of the monomer is more strongly pronounced in solution polymerization. In this case, an analogous effect is observed: the local concentration of the solvent and the monomer inside a polymeric coil can differ from their average concentration in solution. Hence, the relative monomer reactivities will change depending on the polarity of the solvent used in the polymerization. This has been shown first in examples of the copolymerization of four monomer pairs: styrene (S)–methacrylic acid, S–acrylic acid (AA), S–acrylamide and vinylidene chloride-methacrylonitrile, where the monomer reactivity ratios vary considerably as the reaction solvent is varied. However, the copolymers with the same copolymer composition had the same microstructure.^18^

The enrichment or the depletion of a certain monomer around the propagating radical is also dependent on the copolymer composition. In turn, in many cases copolymer chain composition is a function of the chain length.^19–22^ Copolymer composition determines the amount of monomer that is preferentially sorbed around the propagating radical whereas the polarity of the solvent determines which monomer is preferentially sorbed.^23^

A typical example is styrene (S)–acrylic acid (AA) copolymerization: the higher the polarity of the solvent, the more pronounced the difference in monomer reactivity. For example, for copolymerization in bulk *r*_AA_ = 0.15 and *r*_S_ = 0.25,^24^ in DMF *r*_AA_ = 0.05–0.08 and *r*_S_ = 1.03–1.60,^25−27^ in benzene *r*_AA_ = 0.13 and *r*_S_ = 0.30,^27^ and in 1,4-dioxane *r*_AA_ = 0.13 and *r*_S_ = 0.25 for conventional radical copolymerization^28^ and *r*_AA_ = 0.27 and *r*_S_ = 0.72 for a stable free-radical process.^29^

The copolymerization of styrene and acrylic acid *via* a reversible-deactivation radical mechanism has been described in several publications. Moreover, L. Couvreur *et al.*^29^ have reported the use of *N-tert*-butyl-*N*-(1-diethylphosphono-2,2-dimethylpropyl) nitroxide (SG1) and SG1-based alkoxyamine derived from methyl acrylate, CH_3_–O–C(=O)–CH(CH_3_)–SG1, in the controlled stable free-radical polymerization (SFRP) of the above mentioned monomers in 1,4-dioxane at 120 °C. The monomer feed had no effect on either the copolymerization kinetics or molar mass distribution. Due to a three-fold difference in the monomer reactivity ratio, the copolymer composition at some monomer feeds exhibited a slightly pronounced gradient structure. C. Lefay *et al.*^30^ have used the alkoxyamine initiator Blocbuilder™ and SG1 in the synthesis of a gradient copolymer of styrene and acrylic acid of a given molar mass and composition in analogous conditions and have applied the latter as an efficient stabilizer of emulsion polymerization. Later, B. Lessard *et al.*^31^ studied styrene and acrylic acid copolymerization with Blocbuilder™ only and found that the polymerization rates in 1,4-dioxane were strongly affected by the composition of acrylic acid in the feed. The increase of acrylic acid content led to the rise in polymerization rate and broadening of the molar mass distribution. By adding SG1 this effect decreased. In all these studies only low molecular weight controlling agents (nitroxide and alkoxyamine) were used and acrylic acid was less reactive than styrene.

However, recently, O. Borisova *et al.*^32^ showed that when polymeric alkoxyamine based on polyacrylic acid and SG1 is used in styrene–acrylic acid copolymerization in the same solvent, namely 1,4-dioxane, then the relative monomer reactivities change dramatically (*r*_AA_ = 0.94 and *r*_S_ = 0.17). Thus, the authors suggest that this phenomenon is another case demonstrating the effect of preferential monomer sorption, which became possible due to the reversible-deactivation mechanism.

Similarly, an unusual kinetic effect was discovered whereby the significant influence of the RAFT agent on copolymer composition was observed in the synthesis of block copolymers based on poly(dimethylsiloxane), *N*,*N*-dimethyl acrylamide and 2-(*N*-butyl perfluorooctanefluorosulfonamido) ethyl acrylate in α,α,α-trifluorotoluene.^33^

RAFT polymerization was mostly used for the controlled synthesis of either polyacrylic acid or its block-copolymers predominately with poly(*n*-butyl acrylate).^34–37^ In our previous research,^38^ we have applied the RAFT technique to the copolymerization of styrene either with acrylic acid or with *tert*-butyl acrylate for the first time. RAFT copolymerization was carried out both in bulk and in the presence of DMF with a monomer feed containing 85 mol% of styrene and a molar ratio of acrylic acid to DMF equal to 1 : 3. Two low molecular mass RAFT agents, namely dithiobenzoate and symmetrical trithiocarbonate both containing benzyl leaving groups, as well as two analogous oligomeric RAFT agents with polyacrylic acid leaving groups, were used. In that case, we observed that the change in polymer composition depended on the nature of the media and the leaving group of the RAFT agent. However, the question about the relative monomer reactivity ratio as well as the copolymerization features over the broad range of the monomer feed remained open.

Thus, in the present study, we have studied the RAFT copolymerization of styrene and acrylic acid in the presence of DMF using trithiocarbonates and dithiobenzoates with benzyl, polyacrylic acid and polystyrene leaving groups as reversible chain transfer agents. The molar ratio of acrylic acid to DMF was varied from 1 : 1.5 to 1 : 3. A set of copolymers was synthesized and their composition was analyzed. The analysis revealed that the relative monomer reactivities and, hence, copolymer composition at a given monomer feed can be controlled by varying the nature of the RAFT agent. The obtained results confirmed the idea proposed previously that the unusual way that the “bootstrap effect” works when preferential sorption of monomer occurs is due to the use of either a hydrophilic or hydrophobic “living” polymer.

## Results and discussion

### Copolymerization of styrene and acrylic acid mediated by dithiobenzoates

**Fig. 1:**
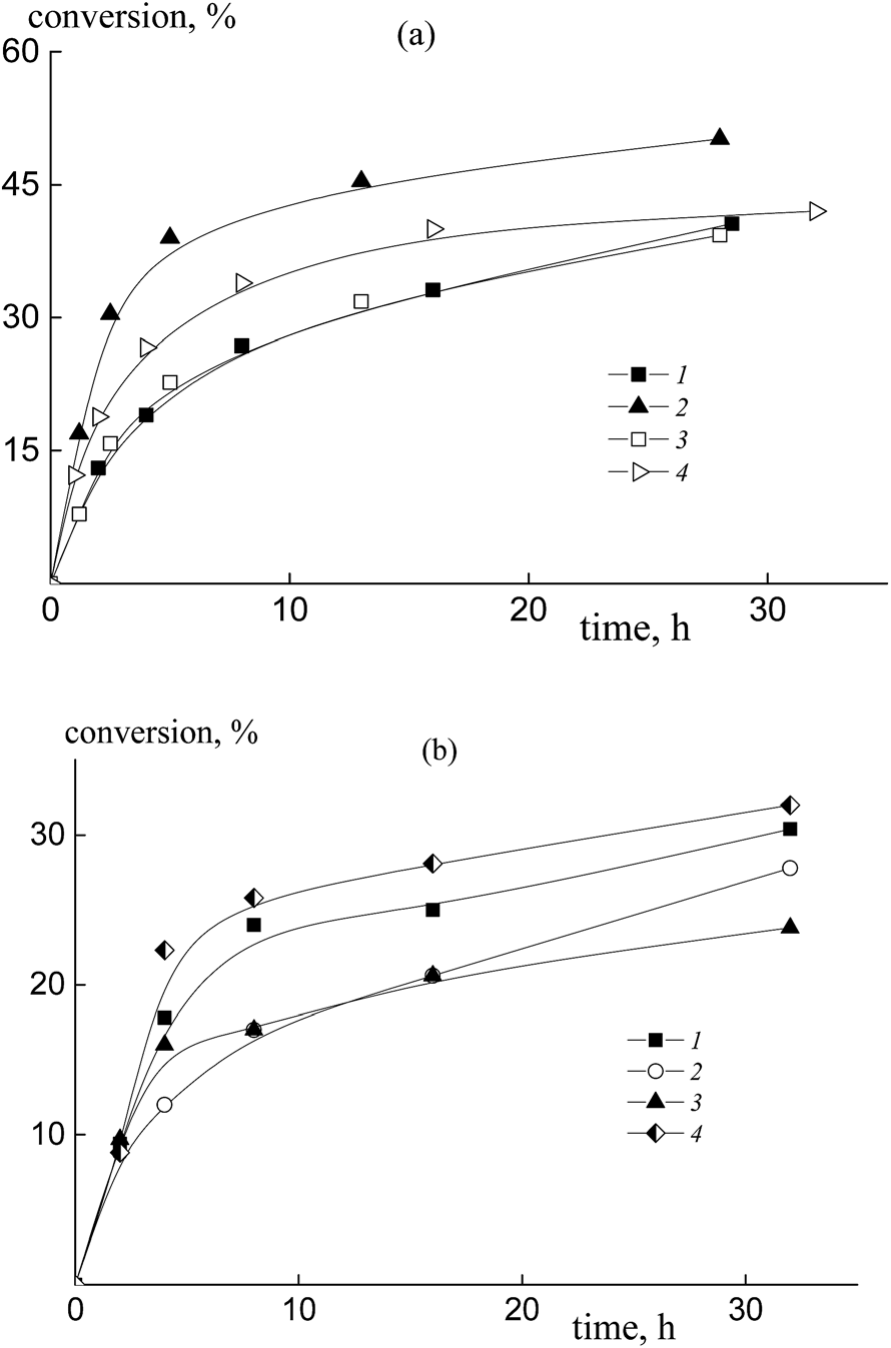
The kinetic plots for copolymerization of styrene and acrylic acid in the presence of DMF, [AIBN]_0_ = 1 × 10^−3^, [BDB]_0_ = [PAADB]_0_ = [PSDB]_0_ = 6 × 10^−3^ mol L^−1^, *T* = 80 °C, and AA : DMF = 1 : 1.5 (a) and 1 : 3 mol (b); (a) *f*_AA_ = 0.5 (1 and 3) and 0.9 (2 and 4), BDB (1 and 2) and PAADB (3 and 4); (b) PSDB, *f*_AA_ = 0.15 (1), 0.30 (2), 0.50 (3) and 0.85 (4).

The copolymerization of styrene and acrylic acid mediated by dithiobenzoates proceeds relatively slowly under the chosen conditions (Fig. 1). At a constant molar ratio of AA to DMF, the increase of AA content in the monomer feed results in an increase of the polymerization rate due to the rise in probability of locating an AA unit at the end of the propagating chain and hence there is an increase in the reactivity of the propagating radical (Fig. 1a). This phenomenon is observed for both RAFT agents, benzyl dithiobenzoate, BDB, (curves 1 and 2) and polyacrylic acid dithiobenzoate, PAADB, (curves 3 and 4), independently from the overall monomer concentration (see Table S1, ESI†). The similar influence of the monomer feed on copolymerization kinetics was described for the SFRP of this monomer pair in the presence of alkoxyamine Blocbuilder™.^31^

There is no visible difference in the kinetics of the equimolar monomer mixture mediated by BDB and PAADB (curves 1 and 3); with an excess of AA in the monomer feed, polymerization proceeds faster when BDB is used (curves 2 and 4). Herein, the conversion of the copolymers produced in the presence of polymeric RAFT agents is referred to as the “grown” copolymer (see the experimental section for details); the *M*_n_ and dispersity of the polymeric RAFT agents are given in Table 4.

The copolymerization of styrene and AA in the presence of polystyrene dithiobenzoate, PSDB, proceeds slowly compared to the systems discussed above (Fig. 1b). However, in this case we have increased the molar ratio of DMF to AA twice, and hence decreased the total monomer concentration (see Table S1, ESI†).

It is clear that the monomer feed has no visible effect on the initial polymerization kinetics, while in the range of middle conversions the polymerization rate first decreases and then increases with the rise in AA content in the initial monomer mixture. This result suggests that the relative monomer reactivities differ for the systems containing BDB, PAADB and PSDB.

**Fig. 2:**
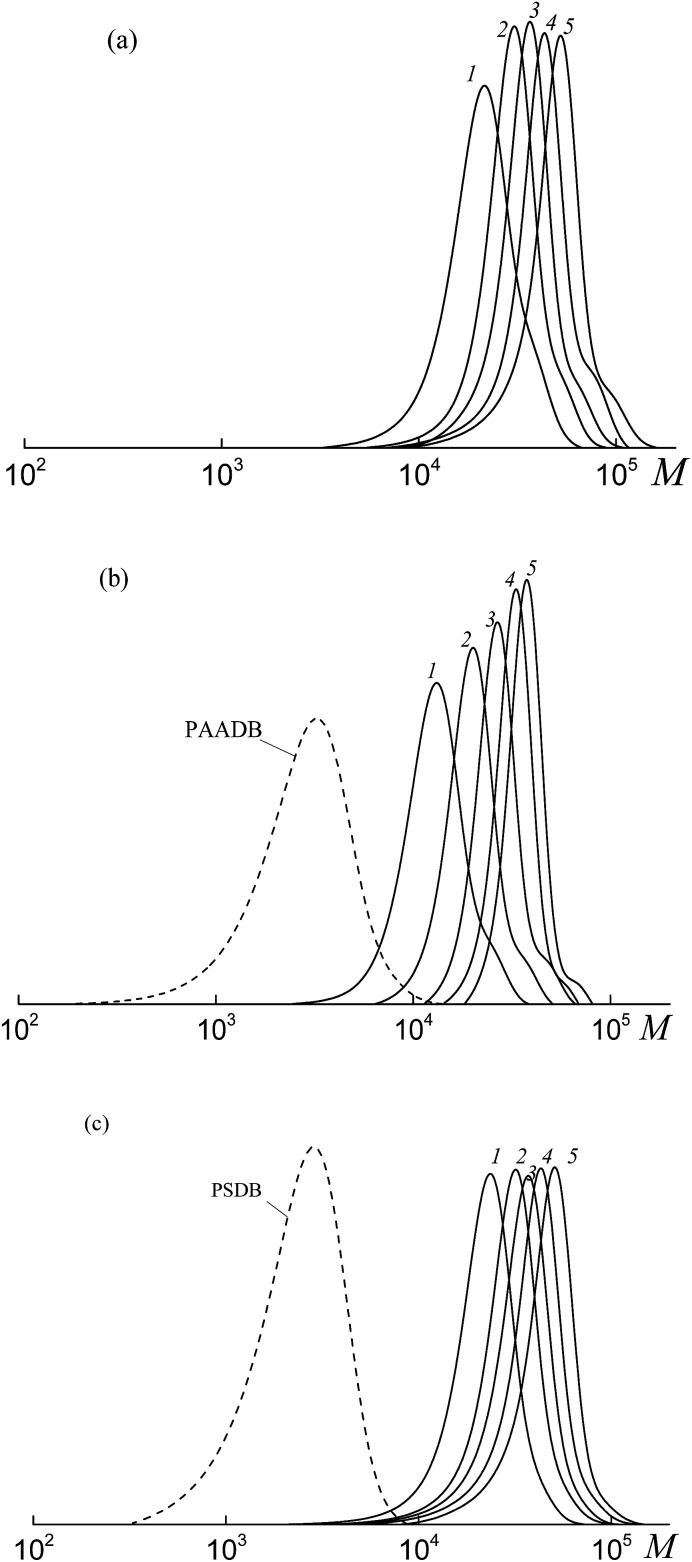
The SEC curves normalized to the unit area for the copolymers formed from an equimolar monomer mixture of styrene and AA in the presence of BDB (a), PAADB (b) and PSDB (c); AA : DMF = 1 : 1.5 (a and b) and 1 : 3 mol (c), [AIBN]_0_ = 1 × 10^−3^, [BDB]_0_ = [PAADB]_0_ = [PSDB]_0_ = 6 × 10^−3^ mol L^−1^, *T* = 80 °C. (a) Conversions: 13.0 (1), 19.0 (2), 26.8 (3), 33.1 (4) and 40.6% (5); (b) conversions: 7.8 (1), 15.8 (2), 22.7 (3), 31.8 (4) and 39.3% (5); (c) conversions: 9.7 (1), 16.0 (2), 17.0 (3), 20.6 (4) and 23.8% (5).

All the investigated systems exhibit the features of controlled radical polymerization. A typical example is shown in Fig. 2, where the SEC traces of the methylated copolymers formed in the copolymerization of the equimolar monomer mixture mediated by BDB, PAADB and PSDB are presented. The molecular weight characteristics of the initial PAADB and PSDB are described in the experimental section. In all cases, the initial RAFT agent is rapidly consumed and the SEC curves shift to the region of high molecular weight as the monomer conversion progresses. The copolymers formed are characterized by narrow MWD.

Thus, it might be concluded that independently from the chemical nature of the leaving group all the studied dithiobenzoates are efficient RAFT agents for the copolymerization of styrene and acrylic acid. When BDB is used, a random or gradient copolymer is formed depending on the initial monomer feed (see below). The use of PAADB or PSDB according to the “living” mechanism results in the synthesis of block copolymers, in which the first block is formed by a polymeric RAFT agent and the second by growing random copolymer PAA-*block*-P(AA-*co*-S).

It is worth noting that when BDB and PAADB are applied in the synthesis, the shoulder is observed on the high molecular weight region of the SEC curves, which is more pronounced for the systems containing BDB. Moreover, this shoulder is absent on the chromatograms of the copolymers formed in the presence of PSTB. The analogous situation is observed for other investigated monomer feeds (Fig. S1 and S2, ESI†). This phenomenon (formation of a certain amount of by-product with a higher molecular mass than the major product) is typical for RAFT polymerization in the presence of dithiobenzoates. This may come from side reactions – square-law termination of propagating radicals or their cross-termination with intermediate radicals.

The suppression of the side reactions may result from the decrease of either propagating radical concentrations or their rate coefficients, *i.e.* reactivity. We should note that in all of the systems, the AIBN and RAFT agent concentrations are the same while the overall monomer concentration differs by 1.4–1.6 times. However, the latter has no influence on the steady-state concentration of propagating radicals. Thus, we propose that the relative monomer reactivities may change in these systems leading to a change in the nature of the terminal monomer unit in the propagating radicals and therefore their reactivity. This assumption is in accordance not only with the presence/absence of the shoulder on the chromatograms of the copolymers formed from the same monomer feed in the presence of dithiobenzoates with different leaving groups, but also with differences in the polymerization kinetics in these systems.

**Fig. 3:**
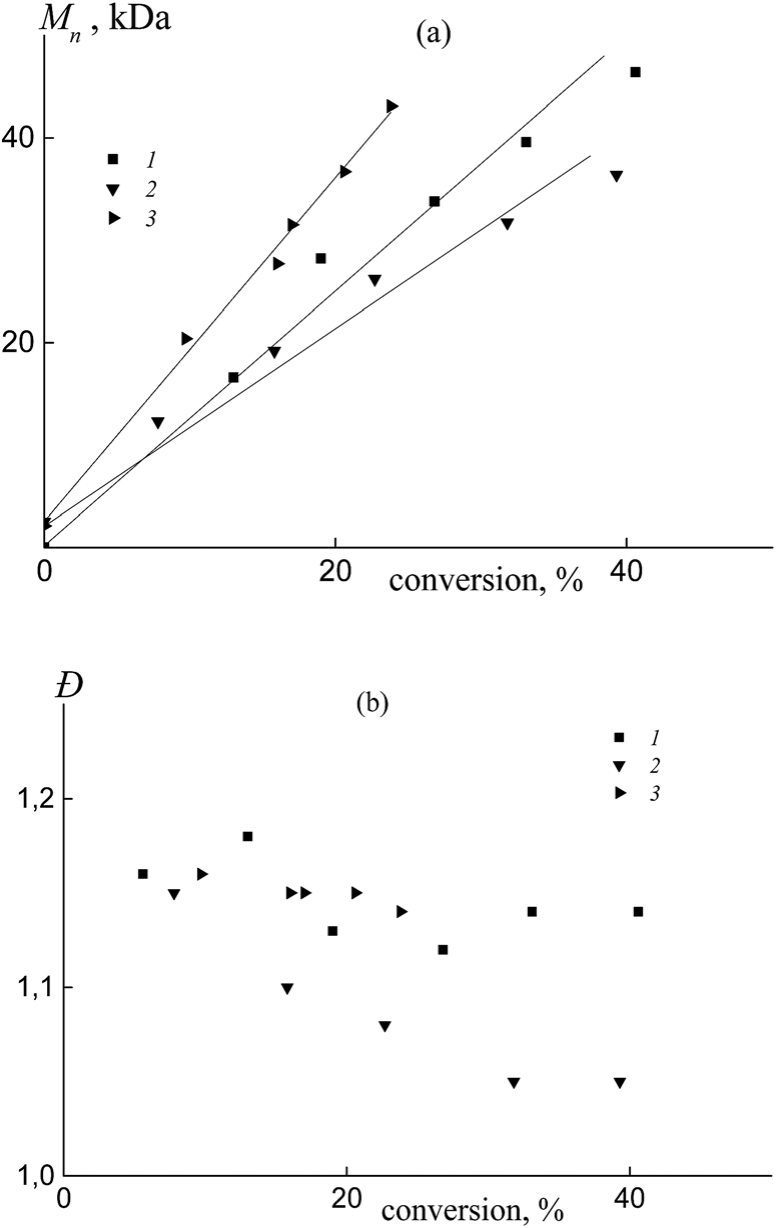
The dependence of *M*_n_ (a) and *Đ* (b) from the overall monomer conversion for the copolymers formed from an equimolar monomer mixture of styrene and AA in the presence of BDB (1), PAADB (2) and PSDB (3); AA : DMF = 1 : 1.5 (1 and 2) and 1 : 3 mol (3), [AIBN]_0_ = 1 × 10^−3^, [BDB]_0_ = [PAADB]_0_ = [PSDB]_0_ = 6 × 10^−3^ mol L^−1^ and *T* = 80 °C.

In all the investigated systems, the number average molar mass *M*_n_ increases linearly with growth of the overall monomer conversion. Fig. 3a presents the typical linear dependence of *M*_n_*versus* conversion for the copolymers formed from an equimolar monomer mixture in the presence of BDB (1), PAADB (2) and PSDB (3). Notice that the straight lines have different slopes. As was mentioned above, the RAFT agent concentration was kept constant throughout the synthesis and the monomer concentration was equal in the experiments with BDB and PAADB, while it was ∼1.4 times lower in the experiment with PSDB (Table S1, ESI†). Hence, in accordance with the equation *M*_n_ = conversion × [M]_0_/[RAFT agent]_0_ we should expect the same slope for dependence (1) and (2) and a smaller slope for dependence (3). The contradiction of the theoretical and experimental results can be explained only by a difference in copolymer composition. Indeed, the average molecular masses were determined using an RI detector and PS calibration without recalculation for the copolymers due to the unknown values of the Mark–Houwink–Sakurada parameters.

All of the synthesized copolymers were characterized by low values of dispersity *Đ* (Fig. 3b). Analogous results were obtained for the copolymers obtained from other monomer feeds (Fig. S3, ESI†).

**Fig. 4:**
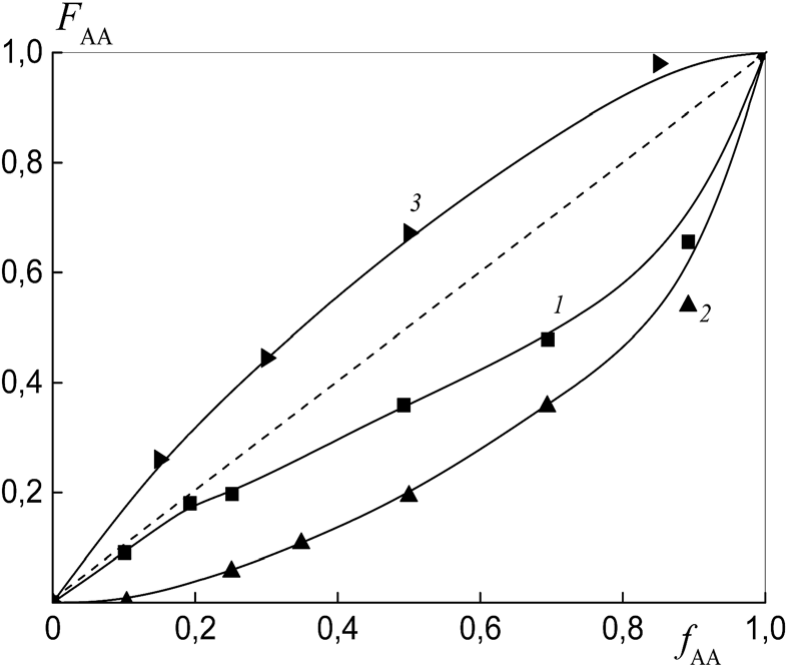
The dependence of the molar fraction of AA in the “grown” copolymer (*F*_AA_) from the molar fraction of AA in the monomer feed (*f*_AA_) for the copolymers formed at overall monomer conversions less than 10% in the presence of BDB (1), PAADB (2) and PSDB (3); AA : DMF = 1 : 1.5 (1 and 2) and 1 : 3 mol (3), [AIBN]_0_ = 1 × 10^−3^, [BDB]_0_ = [PAADB]_0_ = [PSDB]_0_ = 6 × 10^−3^ mol L^−1^ and *T* = 80 °C.

To estimate the relative monomer reactivities, the copolymers were synthesized from different monomer mixtures at conversions below 10%. When the polymeric RAFT agents were used, the composition of the “grown” copolymers was evaluated by excluding the contribution of the polymeric RAFT agent to the composition of the gross copolymer according to the procedure described in detail in the ESI† (Experimental procedures). The results are presented in Fig. 4 as the dependence of the (i) AA molar composition in the copolymer in the case of BDB and (ii) AA molar composition in the “grown” copolymer P(AA-*co*-S), *i.e.* excluding the polymeric RAFT agent from the gross composition *versus* the AA composition in monomer feed. It is clear that the copolymer composition differs strongly depending on the chemical nature of the leaving group in dithiobenzoate. The copolymer is always enriched with AA when PSDB is used (curve 3) and the copolymer is always enriched with styrene when PAADB is used (curve 2). Finally, an intermediate case is observed for BDB (curve 1).

Reactivity ratios were estimated using a terminal unit model by a nonlinear least-squares method and by the Fineman–Ross method (Table 1).

**Table 1:** The relative monomer reactivity ratios for the RAFT copolymerization of styrene and AA in DMF mediated by various dithiobenzoates^a^

RAFT agent	Nonlinear least-squares method	Fineman–Ross method
BDB	*r*_AA_ = 0.14 ± 0.01	*r*_AA_ = 0.16 ± 0.10
	*r*_S_ = 1.00 ± 0.01	*r*_S_ = 1.01 ± 0.03
PAADB, *M*_n_ = 2.4 kDa, *Đ* = 1.36	*r*_AA_ = 0.09 ± 0.02	*r*_AA_ = 0.25 ± 0.59
	*r*_S_ = 3.5 ± 1.2	*r*_S_ = 3.3 ± 1.7
PSDB, *M*_n_ = 2.1 kDa, *Đ* = 1.31	*r*_AA_ = 3.3 ± 0.4	*r*_AA_ = 9.1 ± 4.2
	*r*_S_ = 0.72 ± 0.05	*r*_S_ = 1.9 ± 1.8

a Molar ratio AA/DMF = 1/1.5 for BDB and PAADB and 1/3 for PSDB.

The relative reactivity of AA increases over the range of RAFT agents as follows: PAADB < BDB ≪ PSDB. In contrast to a low polarity solvent, such as 1,4-dioxane (*ε* = 2.2), in which the relative reactivity of AA increased in the presence of the alkoxyamine initiator based on polyacrylic acid,^32^ the use of the polar solvent DMF (*ε* = 36.7) leads to an adverse effect. As a result, the content of AA in the copolymer formed in the presence of PAADB decreases even in comparison with the system containing BDB. The opposite effect is caused by the use of PSDB.

In the aforementioned systems polymeric RAFT agents with a similar *M*_n_ have been used, however they differ by the degree of polymerization. Since the chain length of the polymeric RAFT agent may influence the relative reactivities of the monomers, a series of PAADB with various *M*_n_ values were synthesized (PAADB1–PBAADB3, Table 4). The copolymers of AA and styrene were synthesized from different monomer mixtures at conversions below 10% in the presence of PAADB1–PBAADB3; the molar ratio of AA to DMF in these experiments was equal to 1/3.

**Fig. 5:**
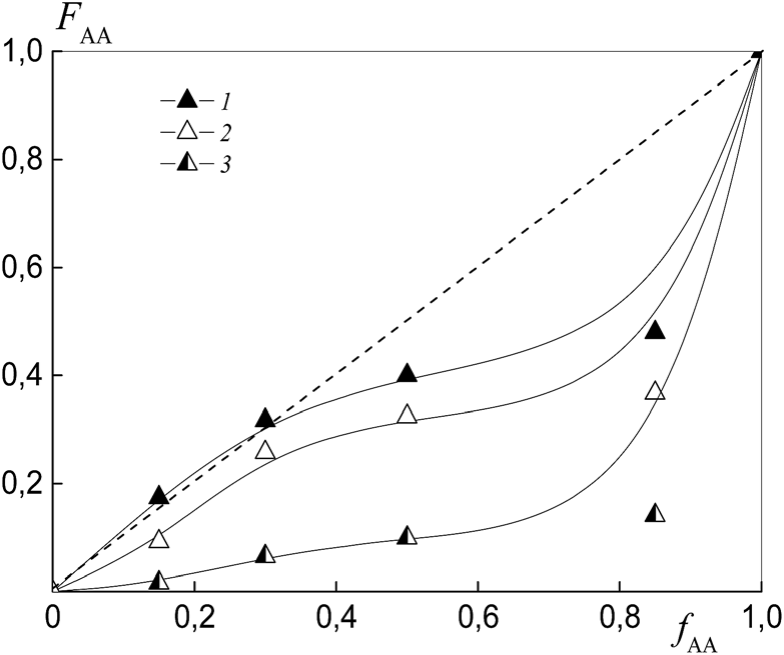
The dependence of the molar fraction of AA in the “grown” copolymer (*F*_AA_) from the molar fraction AA in the monomer feed (*f*_AA_) for the copolymers formed at overall monomer conversions less than 10% in the presence of PAADB1 (1), PAADB2 (2) and PAADB3 (3); AA : DMF = 1 : 3, [AIBN]_0_ = 1 × 10^−3^, [PAADB1]_0_ = [PAADB2]_0_ = [PAADB3]_0_ = 6 × 10^−3^ mol L^−1^ and *T* = 80 °C.

Fig. 5 depicts the dependence of the AA molar composition in the “grown” copolymer P(AA-*co*-S), *i.e.* excluding the polymeric RAFT agent from the gross composition *versus* the AA composition in monomer feed. The copolymer composition is found to depend strongly on the length of the polymeric substituent in dithiobenzoate. The increase in the degree of polymerization in PAATB (from PAATB1 to PAATB3) leads to a rise in the difference between the relative reactivities of the monomers (Table 2). It is worth noting that these experiments were conducted for copolymers obtained at 2, 5 and 7% monomer conversion. Similar data were produced in this range of monomer conversion (Fig. S4, ESI†). This result is similar to the known influence of the concentration of initiator on the relative reactivities of the monomers.^41^ Indeed, the decrease in concentration of the initiator leads to the growth of the *M*_n_ of the copolymer formed, which in turn results in the enhanced difference in relative monomer reactivities.^41^

**Table 2:** Relative monomer reactivity ratios for the RAFT copolymerization of styrene and AA in DMF mediated by PAADB of various molecular weights, molar ratio AA/DMF = 1/3

RAFT agent	Nonlinear least-squares method
PAADB1, *M*_n_ = 3.0 kDa, *Đ* = 1.30	*r*_AA_ = 0.009 ± 0.002
	*r*_S_ = 0.61 ± 0.01
PAADB2, *M*_*n*_ = 5.3 kDa, *Đ* = 1.23	*r*_AA_ = 0.04 ± 0.02
	*r*_S_ = 1.25 ± 0.22
PAADB3, *M*_n_ = 9.0 kDa, *Đ* = 1.16	*r*_AA_ = 0.1 ± 0.07
	*r*_S_ = 8.3 ± 14.3

It may be assumed that replacing the homopolymeric RAFT agents by dithiobenzoates based on copolymers of AA and styrene of various compositions can change the relative monomer reactivities across a wide range. However this subject goes beyond the present research.

**Fig. 6:**
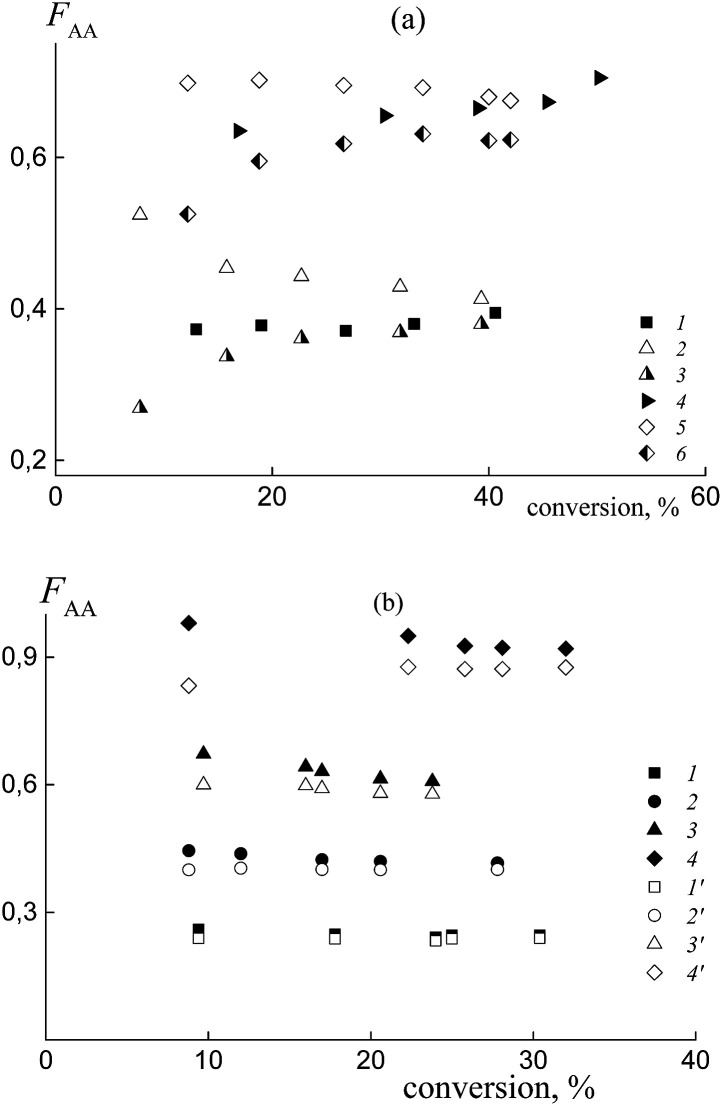
The average molar fraction of AA in the copolymer *versus* the overall monomer conversion for the copolymers synthesized from the monomer mixture containing (a) BDB and PAADB, and (b) PSDB. (a) The content of AA in the feed: 50 (1, 2 and 3) and 90 mol% (4, 5 and 6); BDB (1 and 4) and PAADB (2, 3, 5 and 6); 2 and 5 – the overall copolymer composition, 3 and 6 – the composition of the grown copolymer, AA : DMF = 1 : 1.5. (b) The content of AA in the feed: 15 (1 and 1′), 30 (2 and 2′), 50 (3 and 3′) and 85 mol% (4 and 4′); AA : DMF = 1 : 3; 1–4 – the composition of the grown copolymer, 1′–4′‘ – the overall copolymer composition.

In practice, the average copolymer composition in the investigated systems obtained from a similar monomer feed is different. This result confirms the different relative reactivities of the monomers in copolymerization. Fig. 6a illustrates the dependence of the composition *versus* monomer conversion for the copolymers formed from the mixtures containing BDB (1 and 4) and PAADB (2, 3, 5 and 6). In the last case, the total copolymer composition (2 and 5) and the composition of the grown copolymer, excluding PAADB, (3 and 6) are given. It is observed that the average copolymer composition for the systems containing BDB and PAADB becomes similar at a conversion of ∼40% (1–3 and 4–6). However, at lower monomer conversion the copolymers formed in the presence of PAADB are more enriched with styrene, which is in accordance with the theoretical prediction (Fig. S5, ESI†). For these systems, the molar fraction of AA in the copolymer remains lower in its content in the monomer feed. Fig. 6b presents the composition *versus* conversion for the copolymer grown in the presence of PSDB at different monomer feeds. In all cases, the content of AA in the grown copolymer is higher than that in the initial monomer feed indicating the higher reactivity of AA compared to the systems discussed above.

Note that the difference between the overall copolymer composition and the composition of the grown copolymer is rather small compared to the systems containing PAADB.

So, what may be the reason for the variation in relative monomer reactivity when changing the nature of the leaving group in the RAFT agent?

The RAFT process includes the elementary stages of initiation, chain propagation, reversible addition–fragmentation chain transfer and chain termination. As monomer reactivity is determined by the chain propagation stage, from initial reasoning, we should not expect changes by replacing one RAFT agent with another. However, if the RAFT agent is efficient, as it is in our case, then after the first act of reversible chain transfer of propagating the radical with BDB, PAADB and PSDB three different initiating radicals appear in the system, namely benzyl, polyacrylic acid and polystyrene radicals. Their reactivity in the reaction with monomers may differ due to the solvent effect. For example, if we compare the copolymer prepared *via* copolymerization in bulk and in DMF in the presence of BDB, we find that the former is more enriched with AA units.^38^ This may come from the solvent-induced dissociation of the dimers of carboxylic acids and the formation of H-complexes of DMF and AA, which leads to a decrease in AA reactivity. Another factor related to the effect of preferential solvation is when the polar monomer (AA) is replaced by the polar solvent from the macromolecular coil. The latter factor may become more pronounced when propagating the radical of polyacrylic acid, resulting in an additional decrease in the relative reactivity of AA. In contrast, the preferential solvation of AA occurs when propagation of the radical is based on polystyrene, which results in an increase in the relative reactivity of AA.

If this assumption is reliable, then we should observe an analogous trend when using another class of RAFT agents, namely, trithiocarbonates.

### Copolymerization of styrene and acrylic acid mediated by trithiocarbonates

**Fig. 7:**
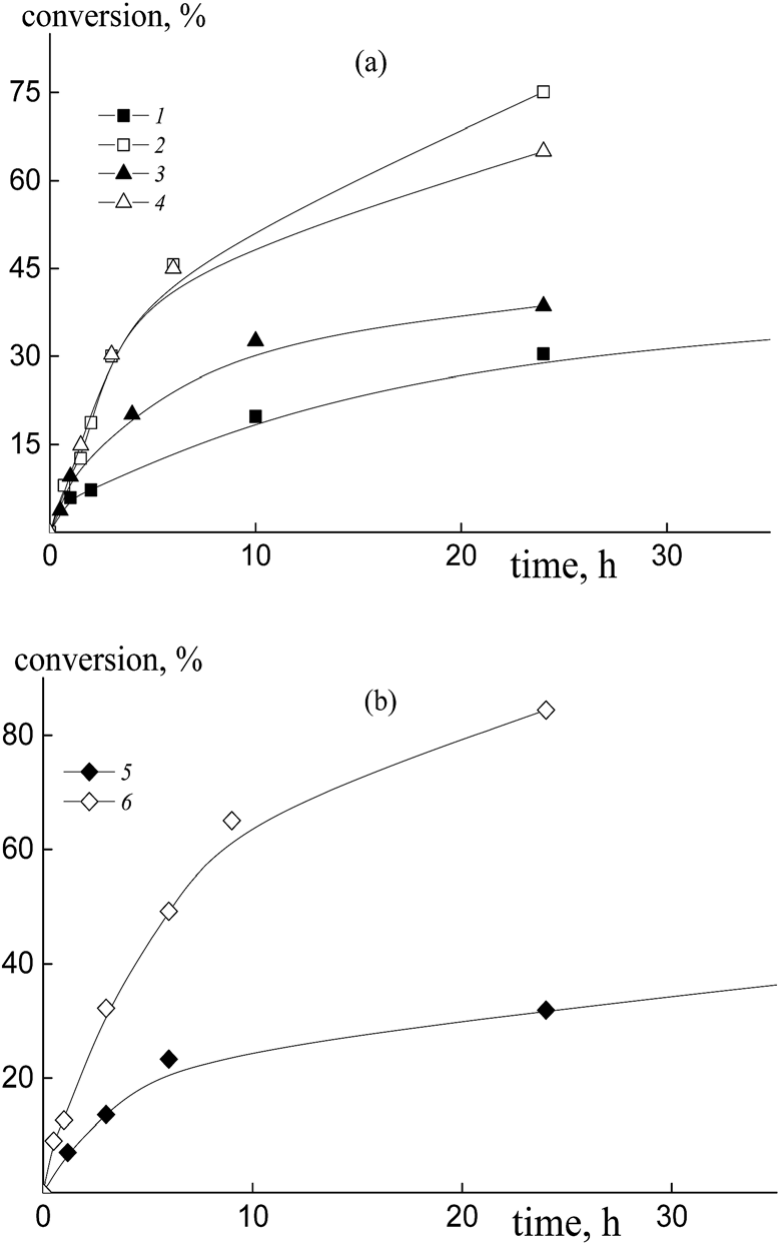
The kinetic plots for the copolymerization of styrene and acrylic acid in the presence of DMF, [AIBN]_0_ = 1 × 10^−3^, [BTC]_0_ = [PAATC]_0_ = [PSTC]_0_ = 6 × 10^−3^ mol L^−1^, *T* = 80 °C, and AA : DMF = 1 : 3 (1, 3 and 5) and 1 : 1.57 mol (2, 4 and 6); *f*_AA_ = 0.5 (1, 3 and 5) and 0.9 (2, 4 and 6), BTC (a – 1 and 2), PAATC (a – 3 and 4) and PSTC (b – 5 and 6).

The copolymerization of styrene and acrylic acid in the presence of trithiocarbonates (Fig. 7, curves 2 and 4–6) proceeds faster under the same conditions than the systems containing dithiobenzoates due to the lower stability of the intermediate radicals^39^ (Fig. 1, curves 2 and 4). The polymerization rate grows with the increase of AA content in the monomer feed independently from the nature of the leaving group of the RAFT agent. Notice that the overall monomer concentration rises only by ∼10% when the AA molar fraction increases from 50 to 90% due to a change in the DMF/AA molar ratio (Table S1, ESI†). Interestingly in the case of trithiocarbonates there is no noticeable difference in the kinetics of the mixtures containing excess AA and mediated by dibenzyl trithiocarbonate, BTC, polyacrylic acid trithiocarbonate, PAATC, or polystyrene trithiocarbonate, PSTC (Fig. 7, curves 2, 4 and 6).

In the case of the equimolar monomer feed, polymerization proceeds faster when PAATC is used (Fig. 7, curves 1 and 3). This is inverse to the kinetics observed for the systems containing dithiobenzoates. Thus, the copolymerization kinetics in the presence of dithiobenzoates and trithiocarbonates have both general and peculiar features.

**Fig. 8:**
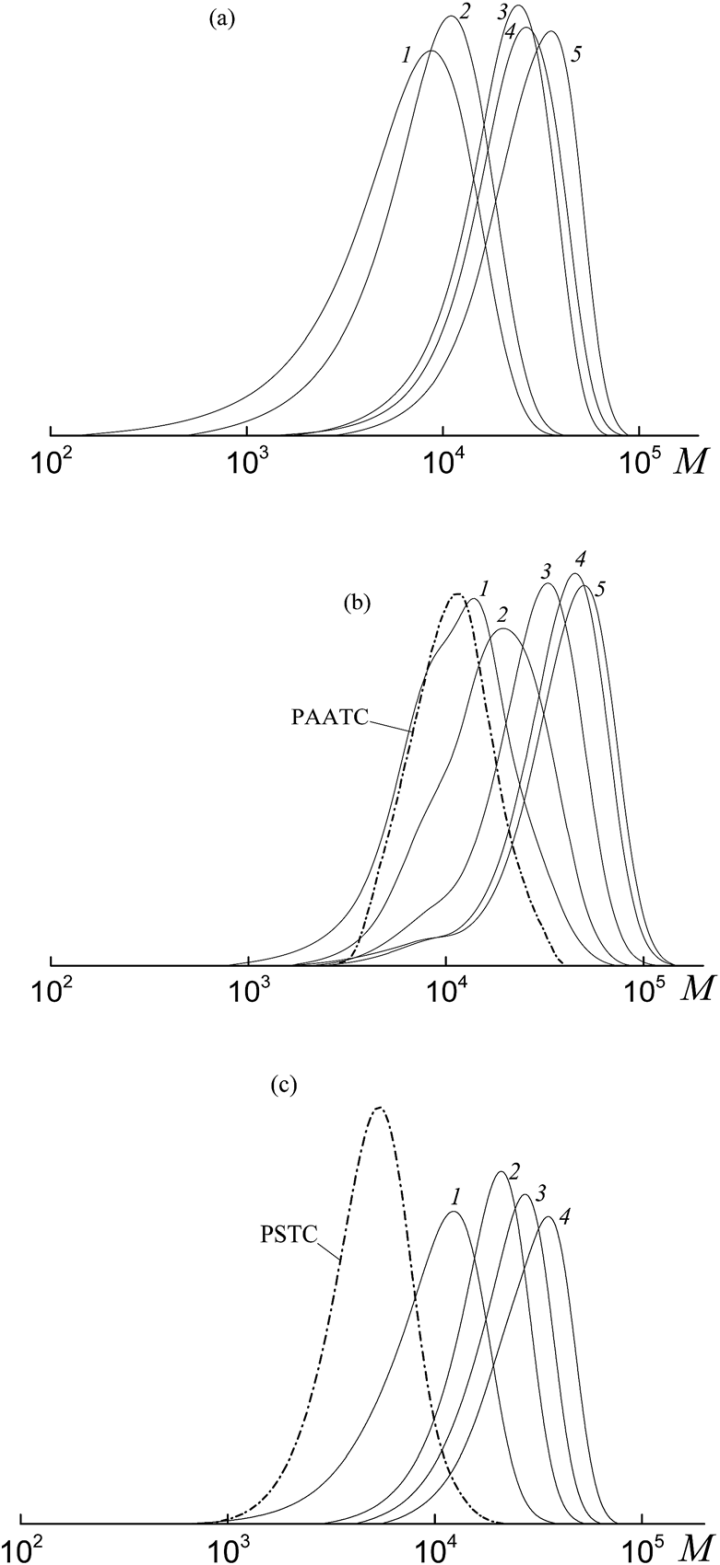
The SEC curves normalized to the unit area for the copolymers formed from an equimolar monomer mixture of styrene and AA in the presence of BTC (a), PAATC (b) and PSTC (c); AA : DMF = 1 : 3 mol (c), [AIBN]_0_ = 1 × 10^−3^, [BTC]_0_ = [PAATC]_0_ = [PSTC]_0_ = 6 × 10^−3^ mol L^−1^ and *T* = 80 °C. (a) Conversions: 5.9 (1), 6.2 (2), 30.4 (3), 36.3 (4) and 51.5% (5); (b) conversions: 3.7 (1), 9.5 (2), 20.1 (3), 32.6 (4) and 38.6% (5); (c) conversions: 7.0 (1), 13.6 (2), 23.3 (3) and 31.9% (4).

The SEC analysis of the methylated copolymers revealed the controlled behavior of the copolymerization mediated by trithiocarbonates (Fig. 8). The molecular weight characteristics of the initial PAATC and PSTC are described in the experimental section. As observed with the growth of monomer conversion, the chromatograms are shifted to the high molecular mass region. However, comparing Fig. 2 and 8, one can discover the poorer control of molecular mass characteristics in the systems containing trithiocarbonates (Fig. 8). Indeed, the MWD of all of the copolymers is broader due to the tail in the low molecular mass region of the SEC curves. Moreover, the PAATC is consumed slowly in the course of the polymerization compared to PAADB; hence the efficiency of the former in reversible chain transfer is less. The increase of AA content in the monomer feed allows the efficiency of PAATC to rise and very slightly affects the efficiency of BTC and PSTC (Fig. S6, ESI†).

**Fig. 9:**
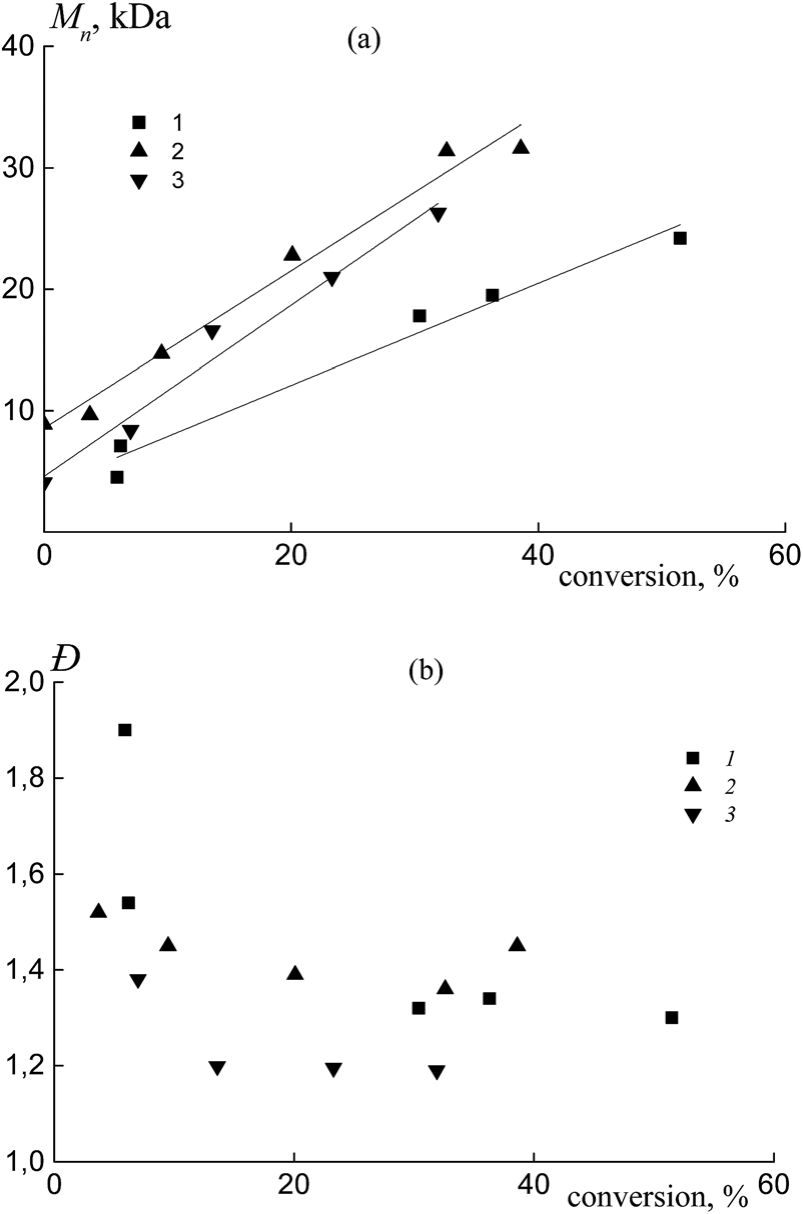
The dependence of *M*_n_ (a) and *Đ* (b) from overall monomer conversion for the copolymers formed from an equimolar monomer mixture of styrene and AA in the presence of BTC (1), PAATC (2) and PSTC (3); AA : DMF = 1 : 3 mol (3), [AIBN]_0_ = 1 × 10^−3^, [BTC]_0_ = [PAATC]_0_ = [PSTC]_0_ = 6 × 10^−3^ mol L^−1^ and *T* = 80 °C.

Independently from the AA content in the monomer feed, the number average molar mass of the copolymer obtained in the presence of BTC, PAATC and PSTC increases linearly with the progress in monomer conversion (Fig. 9a and S7a, ESI†), confirming a “living” mechanism and hence the formation of random/gradient BTC of block-random/block-gradient copolymer PAA-*block*-P(AA-*co*-S)-*block*-PAA or PS-*block*-P(AA-*co*-S)-*block*-PS.

In all cases, the straight lines have different slopes similar to those discussed for the above systems (Fig. 3a). The dispersity *Đ* firstly decreased with monomer conversion and then was kept constant or slightly increased (Fig. 9b and S7b, ESI†). In general, its value is a little higher compared to that of the copolymers that were synthesized using dithiobenzoates (Fig. 3b).

In contrast to dithiobenzoates, when the bifunctional trithiocarbonate R–S–C(=S)–S–R is used in the polymer synthesis, the monomer units are randomly incorporated into macromolecules at one or both sides with respect to the trithiocarbonate group. This leads to the formation of two types of macromolecule (P_*n*_–S–C(=S)–S–R and P_*n*_–S–C(=S)–S–P_*m*_). The period of coexistence of these macromolecules depends on the conversion, the chemical nature of monomers and the leaving group R.^39^ To assess the locus of the trithiocarbonate group in the macromolecule the approach described in detail elsewhere was used.^40^ Briefly, the polymer is heated with a high excess of the radical initiator in an inert solvent over a required period of time. After that, the molecular mass characteristics of the polymer before and upon heating with an excess of initiator are analyzed. When the trithiocarbonate group is located inside the macromolecule P_*n*_–S–C(=S)–S–P_*m*_ the molecular weight of the resultant product appears to be two times lower if P_*n*_ ≈ P_*m*_, or macromolecules with a lower but different MW are formed if P_*n*_ ≠ P_*m*_. If the macromolecule has a structure of P_*n*_SC(=S)SR, then the macromolecules formed after heating with an excess of the radical initiator have nearly the same molecular weight but different functionalities of the end groups. Even when the removal of the trithiocarbonate group is incomplete, the shift of the main peak in the SEC chromatogram makes it possible to draw certain conclusions concerning the symmetry of the trithiocarbonate group locus in the macromolecule.^40^

**Fig. 10:**
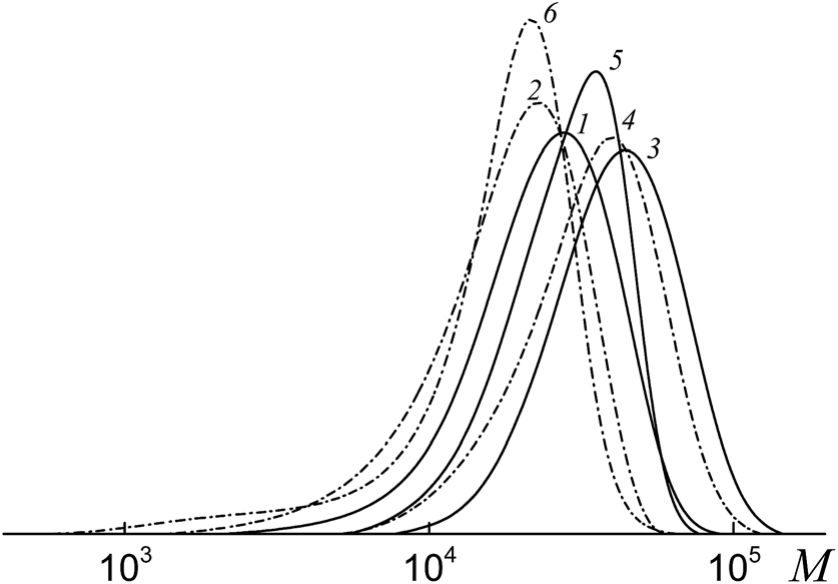
The SEC curves normalized to the unit area for the copolymers formed from an equimolar monomer mixture of styrene and AA in the presence of BTC (1), PAATC (3) and PSTC (5) and the same copolymers after heating with a 100-fold excess of AIBN (2, 4 and 6).

Fig. 10 presents the SEC curves before (curves 1, 3 and 5) and after (curves 2, 4 and 6) thermal treatment with AIBN for the copolymers synthesized from an equimolar monomer mixture of styrene and AA using the bifunctional trithiocarbonates BTC (1), PAATC (2) and PSTC (3).

According to the reasoning given above, comparison of the position of the peaks (curves 1, 3 and 5 *versus* curves 2, 4 and 6) allows one to conclude that in all the copolymers the trithiocarbonate group is located close to the mid-chain, however, when PAATC is used its location is more asymmetric within the chain. Therefore, chain propagation takes place at both sides with respect to the trithiocarbonate group. An analogous trend is observed for the copolymers synthesized from the monomer mixture containing an excess of AA (Fig. S8, ESI†).

All of the above results on copolymerization kinetics and molecular mass distribution allow us to suggest that the relative monomer reactivities in the systems containing trithiocarbonates with various leaving groups may be different and they may also differ from those in the systems containing dithiobenzoates.

**Fig. 11:**
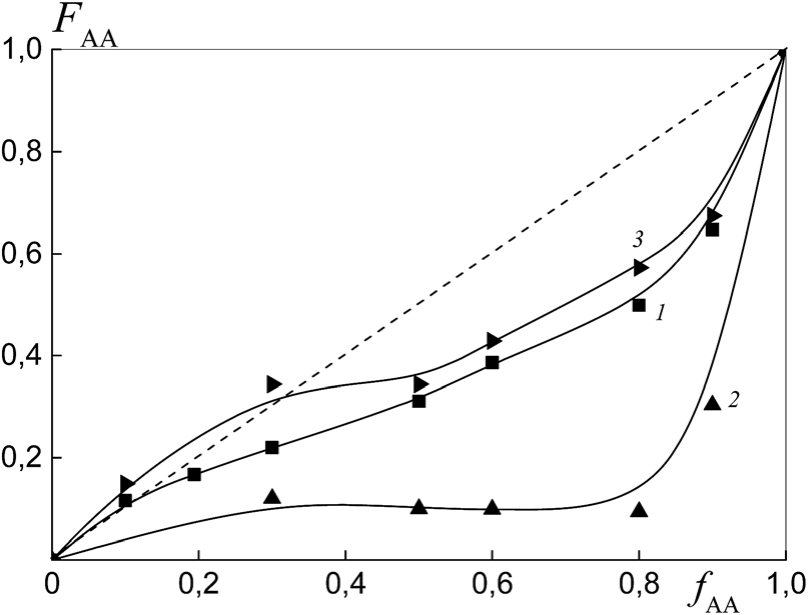
The dependence of the molar fraction of AA in the copolymer (*F*_AA_) from the molar fraction of AA in the monomer feed (*f*_AA_) for the copolymers formed at overall monomer conversions of less than 10% in the presence of BTC (1), PAATC (2) and PSTC (3); AA : DMF = 1 : 3 mol, [AIBN]_0_ = 1 × 10^−3^, [BTC]_0_ = [PAATC]_0_ = [PSTC]_0_ = 6 × 10^−3^ mol L^−1^ and *T* = 80 °C.

Fig. 11 presents the dependence of the AA molar part in the copolymer *versus* its molar part in the monomer feed for the copolymers synthesized from different monomer mixtures at conversions below 10%. As expected, the relative monomer reactivities differ depending on the chemical nature of the leaving group in trithiocarbonate. The copolymer is always enriched with styrene when PAATC is used (curve 2).

The azeotropic point is observed when BTC (curve 1) and PSTC (curve 3) are used, and its value differs for these systems. One can see that the use of PSTC results only in the decrease of the difference between styrene and AA reactivity, in contrast to when PSTB is used (Fig. 4, curve 3).

The reactivity ratios were estimated using a terminal unit model by a nonlinear least-squares method and by the Fineman–Ross method (Table 3). As in the systems discussed above, the relative activity of AA increases with the range of the leaving groups of the RAFT agents as follows: PAA < benzyl < PS, *i.e.* in the range of the RAFT agents PAATC < BTC < PSTC.

**Table 3:** Relative monomer reactivity ratios for the RAFT copolymerization of styrene and AA in DMF mediated by various trithiocarbonates

RAFT agent	Nonlinear least-squares method	Fineman–Ross method
BTC	*r*_AA_ = 0.08 ± 0.01	*r*_AA_ = 0.20 ± 0.18
	*r*_S_ = 0.85 ± 0.03	*r*_S_ = 0.71 ± 0.04
PAATC, *M*_n_ = 8900, *Đ* = 1.34	*r*_AA_ = 0.08 ± 0.04	*r*_AA_ = 0.26 ± 0.22
	*r*_S_ = 3.03 ± 1.78	*r*_S_ = 2.39 ± 0.51
PSTC, *M*_n_ = 4100, *Đ* = 1.20	*r*_AA_ = 0.11 ± 0.01	*r*_AA_ = 0.10 ± 0.10
	*r*_S_ = 0.54 ± 0.03	*r*_S_ = 0.52 ± 0.02

**Fig. 12:**
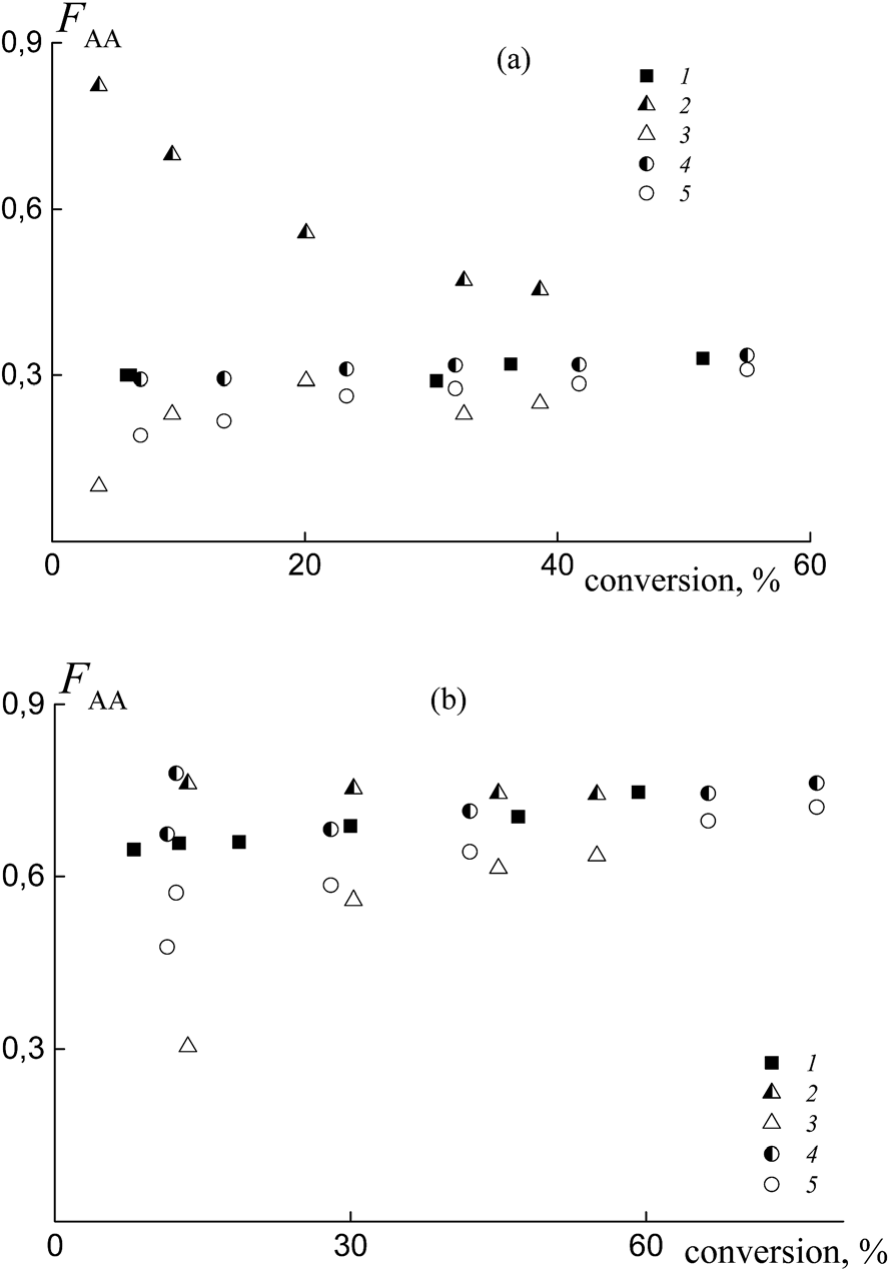
The average molar fraction of AA in the copolymer *versus* overall monomer conversion for the copolymers synthesized from the monomer mixture containing 50 (a) and 90 mol% (b) of AA in the presence of BTC (1), PAATC (2 and 3) and PSTC (4 and 5); AA : DMF = 1 : 3 (a) and 1 : 1.57 mol (b); 2 and 4 – the overall copolymer composition, 3 and 5 – the composition of the grown copolymer.

The experimental data on the average copolymer composition obtained from two monomer feeds (50 and 90 mol% of AA) are given in Fig. 12. It is seen that for the equimolar monomer feed the overall copolymer composition differs for the systems containing BTC (Fig. 12a, curve 1) and PAATC (curve 2), while it is similar for the systems containing BTC and PSTC (curve 4). For the grown copolymers (excluding the contribution of PAATC or PSTC), they are more enriched with styrene than the copolymers formed in the presence of BTC with up to 15–20% of monomer conversion; thereafter the difference is diminished (curves 3 and 5).

With an increase of AA in the monomer feed, the trend in conversion dependence of the copolymer composition remains similar (Fig. 12b). The overall copolymer composition for the systems containing BTC (curve 1) and PSTC (curve 4) is similar and slightly differs for the system containing PAATC (curve 2). The composition of the grown copolymers differs from the copolymers synthesized in the presence of BTC. This difference is pronounced for up to ∼60% of monomer conversion (curves 3 and 5). Thus, trithiocarbonates with different leaving groups also allow the relative monomer reactivities to be governed.

## Experimental

### Materials and polymer synthesis

RAFT agents – dibenzyl trithiocarbonate, BTC, and benzyl dithiobenzoate, BDB – were synthesized and characterized as described elsewhere.^42,43^ Before its use, AIBN was recrystallized from anhydrous methanol. Directly before their use, the monomers, styrene and acrylic acid, and the solvent, *N*,*N*-dimethylformamide, DMF, were distilled under reduced pressure. 1,4-Dioxane was distilled; THF was distilled over KOH.

Four polymeric RAFT agents, namely polystyrene trithiocarbonate, PSTC, polystyrene dithiobenzoate, PSDB, polyacrylic acid trithiocarbonate, PAATC, and polyacrylic acid dithiobenzoate, PAADB, were synthesized. The detailed procedures of their synthesis are presented in the ESI (see experimental procedures); their molar mass characteristics are listed in Table 4.

**Table 4:** Polymerization conditions and molecular weight characteristics of the synthesized polymers

Sample	[RAFT], mol L^−1^	[AIBN], mol L^−1^	*M*_n_ × 10^−3^, kDa	*Đ*
PSTC	BTC, 0.2	10^−2^	4.1	1.20
PSTB	BDB, 0.2	10^−2^	2.1	1.31
PAATC	BTC, 0.1	10^−3^	8.9	1.34
PAADB	BDB, 0.1	10^−3^	2.4	1.36
PAADB1	BDB, 0.1	10^−3^	3.0	1.30
PAADB2	BDB, 0.1	10^−3^	5.3	1.23
PAADB3	BDB, 0.1	10^−3^	9.1	1.16

The reaction mixtures for copolymerization were prepared by dissolving the required amount of the initiator AIBN and the RAFT agent in the freshly distilled monomer mixture containing DMF. In all of the experiments except the systems with 90 mol% of AA and 10 mol% of styrene, the molar ratio of AA and DMF was equal to 1 : 3 or 1 : 1.5; the exact value is given in each experiment in the text. Solutions were poured into the ampoules, degassed by three freeze-pump-thaw cycles and sealed. The ampoules were immersed into a water bath which was pre-heated at 80 °C for the required time. Then, the samples were cooled in liquid nitrogen; the polymers were dissolved in a ten-fold excess of 1,4-dioxane and dried several times by lyophilization under a vacuum. Monomer conversion was determined gravimetrically.

When BDB and BTC were used, the monomer conversion (%) was calculated as (*m*_pol_/*m*_mon_) × 100, where *m*_pol_ is the weight of the copolymer (g) determined after lyophilization and *m*_mon_ is the weight of AA and styrene (g) taken for copolymerization. When the polymeric RAFT agent was used (PAADB, PSDB, PAATC or PSTC), monomer conversion (%) was determined as ((*m*_pol_ − *m*_polyRAFT_)/*m*_mon_) × 100, where *m*_polyRAFT_ is the weight of the polymeric RAFT agent (g) which was used in polymerization.

To estimate the locus of the trithiocarbonate group in the macromolecules synthesized using BTC, PSTC or PAATC, a polymer solution (1 wt%) in 1,4-dioxane containing 0.1 mol L^−1^ AIBN was prepared. After degassing and sealing, the ampoule with the mixture was immersed into the thermostat bath which had been pre-heated at 80 °C for 24 h. Then, the polymer was dried by lyophilization under vacuum from benzene solution and analyzed by SEC.

### Instrumentation

For size exclusion chromatography (SEC), the polymers were modified by methylation of the carboxylic acid groups using diazomethane.

The molecular weight characteristics of the polymers were studied by SEC. The SEC measurements were performed in THF at 40 °C with a flow rate of 1.0 mL min^−1^ using a Shimadzu liquid chromatograph equipped with a refractive index and UV-detectors and two columns packed with styragel with pore dimensions of 10^4^ and 10^5^ Å. The SEC system was calibrated using narrow dispersed linear polystyrene standards. Calculations were carried out using “LCsolution” software.

The composition of the synthesized copolymers was determined by the conductometric titration^44^ of the carboxylic groups with a 0.1 M potassium hydroxide methanol solution using a high-frequency titrator TV-6L1 (Russia). A given amount of the copolymer was dissolved in THF and KOH solution was added dropwise. From the weight of the acidic groups determined at the intersection of the curves, the average composition of the copolymer was calculated according to the procedure described in detail in the ESI.† When polymeric RAFT agents were used in the copolymer synthesis, the molar fraction of acrylic acid in the “grown” copolymer was calculated from the gross amount of acrylic acid in the copolymer and total monomer conversion. Examples of titration curves and the detailed procedure for calculation of the molar fraction of acrylic acid in the “grown” copolymer produced in the presence PAA and PS-based polymeric RAFT agents is given in the ESI (Experimental procedures).

## Conclusions

The main aim of the present research was to demonstrate the comparative analysis of relative monomer reactivities in the RAFT-based copolymerization of polar and low polar monomers in a polar solvent under the action of RAFT agents with various chemical natures. Similar effects are discovered for both dithiobenzoates and trithiocarbonates with different leaving groups. The relative monomer reactivities in the RAFT copolymerization of styrene and acrylic acid in a solution of *N*,*N*-dimethylformamide vary upon changing the leaving group in the RAFT agent (benzyl, polystyryl or polyacrylic acid). Hydrophilic polymeric RAFT agents enhance the difference in the monomer activities compared to low molecular weight RAFT agents, while hydrophobic polymeric RAFT agents exhibit the opposite effect: they either increase the reactivity of acrylic acid or diminish the difference in reactivity of both monomers. Solvent effect (copolymerization in bulk) also has an influence on the relative monomer activities.^38^

Our preliminary results on the RAFT copolymerization of styrene and acrylic acid in 1,4-dioxane, as well as the RAFT copolymerization of butyl acrylate and acrylic acid in bulk and with solvents of various polarities, confirm the possibility of “tuning” the monomer unit distribution in the macromolecule by changing the nature of the solvent and RAFT-agent.

## Conflicts of interest

There are no conflicts to declare.

## Supplementary Material

RA-008-C8RA00048D-s001
