# Supplementary material for: Control over the relative reactivities of monomers in RAFT copolymerization of styrene and acrylic acid
Source: RSC Adv. 2018 Apr 18;8(26):14300–10. doi: 10.1039/c8ra00048d (PMC9080008; doi:10.1039/c8ra00048d)

Electronic Supplementary Material (ESI) for RSC Advances.
This journal is © The Royal Society of Chemistry 2018

Supporting Information for

Control over the relative reactivities of monomers in RAFT copolymerization of styrene and acrylic acid

E. V. Chernikova,[a,*] S. D. Zaitsev,[b,*] A. V. Plutalova[a], M. Z. Bekanova[a], O. S. Zotova[b], and D. V. Vishnevetsky[a]

[a]*Polymer Department, Faculty of Chemistry, Lomonosov Moscow State University, Leninskie gory 1/3, Moscow, 119991, Russian Federation , Fax: +7 495 9397104, Tel: +7 495 9395406;*

[b] *Nizhni Novgorod State University, pr. Gagarina, 23, Nizhni Novgorod, 603950, Russian Federation,*

E-mail: chernikova_elena@mail.ru, szay@inbox.ru

Table of Contents

Experimental Procedures........3

Synthesis of homopolymers........3

Synthesis of copolymers........3

Table S1. Styrene/acrylic acid mixtures for copolymer synthesis.........3

Determination of copolymer composition via conductometric titration........4

Fig. S1. The SEC curves normalized by unit area for copolymers formed from mixture of styrene (10 mol. %) and AA (90 mol. %) in the presence of BDB (a) and PAADB (b). AA : DMF = 1 : 1.5, $[AIBN]_0 = 1\times10^{-3}$, $[BDB]_0 = [PAADB]_0 = 6\times10^{-3}$ mol/L,T = 80°C. (a) Conversions: 16.9 (*1*), 30.4 (*2*), 39.0 (*3*), 45.4 (*4*), and 50.2 % (*5*); (b) conversions: 12.2 (*1*), 18.8 (*2*), 26.6 (*3*), 33.9 (*4*), and 40.0 % (*5*)........7

Fig. S2. The SEC curves normalized by unit area for copolymers formed from different monomer mixtures of styrene and AA in the presence of PSDB, AA : DMF = 1 : 3 mole, $[AIBN]_0 = 1\times10^{-3}$, $[PSDB]_0 = 6\times10^{-3}$ mol/L, T = 80°C. (a) f_{AA} = 0.15, conversions: 9.4 (*1*), 17.8 (*2*), 24.0 (*3*), 25.0 (*4*), and 30.4 % (*5*); (b) f_{AA} = 0.30, conversions: 8.8 (*1*), 12.0 (*2*), 17.0 (*3*), 20.6 (*4*), and 27.8 % (*5*); (c) f_{AA} = 0.85, conversions: 8.8 (*1*), 22.3 (*2*), 25.8 (*3*), 28.1 (*4*), and 32.0 % (*5*)........8

Fig. S3. Dependences of M_n (a) and M_w/M_n (b) from overall monomer conversion for copolymers formed from different monomer mixture of styrene and AA in the presence of BDB (*1*), PAADB (*2*), and PSDB (*3*–*5*), f_{AA} = 0.15 (*3*), 0.3 (*4*), 0.85 (*5*), 0.9 (*1*, *2*), AA :

DMF = 1 : 1.5 (*1*, *2*) and 1 : 3 mole (*3*–5), $[AIBN]_0 = 1\times10^{-3}$, $[BDB]_0 = [PAADB]_0 = [PSDB]_0 = 6\times10^{-3}$ mol/L, T = 80°C..9

Fig. S4. Dependence of molar fraction of AA in grown copolymer (F_{AA}) from molar fraction AA in monomer feed (f_{AA}) for the copolymers formed in the presence PAADB1 (a), PAADB2 (b), and PAADB3 (c) at overall monomer conversion 2 (*1*), 5 (*2*), and 7 % (*3*); AA : DMF = 1 : 3, $[AIBN]_0 = 1\times10^{-3}$, $[PAADB1]_0 = [PAADB2]_0 = [PAADB3]_0 = 6\times10^{-3}$ mole/L, T = 80°C. 10

Fig. S5. The instantaneous diad composition $A_{AA}A_{AA}$ (*1*, *3*, *5*) and $A_{AA}A_S$ (*2*, *4*, *6*) calculated according to the terminal unit model for equimolar monomer mixture of AA and styrene using r_{AA} and r_S determined for the copolymerizations mediated by BDB (*1*, *2*), PAADB (*3*, *4*), and PSDB (*5*, *6*). 12

Fig. S6. The SEC curves normalized to the unit area for copolymers formed from mixture of styrene (10 mol. %) and AA (90 mol. %) in the presence of BTC (a), PAATC (b), and PSTC (c); AA : DMF = 1 : 1.57 mole (c), $[AIBN]_0 = 1\times10^{-3}$, $[BTC]_0 = [PAATC]_0 = [PSTC]_0 = 6\times10^{-3}$ mole/L, T = 80°C. (a) Conversions: 8.0 (*1*), 12.6 (*2*), 18.7 (*3*), 30.0 (*4*), 47.0 (*5*), and 79.2 % (*6*); (b) conversions: 13.5 (*1*), 30.3 (*2*), 45.0 (*3*), and 65.0 % (*4*); (c) conversions: 11.4 (*1*), 12.3 (*2*), 28.0 (*3*), 47.0 (*4*), and 79.2 % (*5*). 13

Fig. S7. Dependence of M_n (a) and M_w/M_n (b) from overall monomer conversion for copolymers formed from mixture of styrene (10 mol. %) and AA (90 mol. %) in the presence of BTC (*1*), PAATC (*2*), and PSTC (*3*); AA : DMF = 1 : 1.57; $[AIBN]_0 = 1\times10^{-3}$, $[BDB]_0 = [PAADB]_0 = [PSDB]_0 = 6\times10^{-3}$ mole/L, T = 80°C. 14

Fig. S8. The SEC curves normalized to the unit area for copolymers formed from mixture of styrene (10 mol. %) and AA (90 mol. %) in the presence of BTC (*1*), PAATC (*3*), and PSTC (*5*) and the same copolymers after heating with the 100-fold excess of AIBN (*2*, *4*, *6*). 15

Experimental Procedures

Synthesis of homopolymers

Polystyrene trithiocarbonate (PSTC), polystyrene dithiobenzoate (PSDB), polyacrylic acid trithiocarbonate (PAATC) and polyacrylic acid dithiobenzoate (PAADB) were synthesized via the RAFT polymerization using dibenzyl trithiocarbonate (BTC) and benzyl dithiobenzoate (BDB) as RAFT-agents and AIBN as a radical initiator. For PSTC and PSDB syntheses, 0.580 g of BTC (0.2 mole/L), 0.0164 g of AIBN (10^{-2} mole/L) and 0.488 g of BDB (0.2 mole/L), 0.0164 g of AIBN (10^{-2} mole/L), respectively were dissolved in 10 ml of the freshly distilled styrene. For PAATC and PAADB syntheses, 0.290 g of BTC (0.1 mole/L), 0.0016 g of AIBN (10^{-3} mole/L) and 0.244 g of BDB (0.1 mole/L), 0.0016 g of AIBN (10^{-3} mole/L), respectively were dissolved in 10 ml of the freshly distilled styrene. Then, standard manipulations were used (see EXPERIMENTAL).

Synthesis of copolymers

Samples for RAFT copolymerization styrene and acrylic acid were prepared through the dissolution of calculated amounts of AIBN, RAFT agents and DMF in a monomer mixture of the desired composition (Table S1).

Table S1. Styrene/acrylic acid mixtures for copolymer synthesis.

RAFT agent	f_{AA}	[DMF]/[AA]	V_{AA}, mL	V_S, mL	V_{DMF}, mL	[M], mol/L
BDB,	0.5	1.5	6.9	11.5	11.6	6.7
PAADB,	0.9	1.5	10.4	1.9	17.6	5.6
PSDB	0.15	3	2.2	20.5	7.3	7.0
	0.30	3	2.4	9.4	8.2	5.9
	0.5	3	6.6	11.1	22.4	4.8
	0.85	3	6.4	1.9	21.7	3.7
BTC,	0.1	3	0.7	10.3	2.3	7.5
PAATC,	0.3	3	1.0	4.0	3.5	5.9
PSTC	0.5	3	3.4	9.8	11.6	4.8
	0.6	3	2.0	2.3	7.0	4.4
	0.8	3	5.5	2.3	11.6	3.8
	0.9	1.57	9.9	1.9	16.7	5.5

Determination of copolymer composition via conductometric titration

The typical conductometric curves are given below for copolymers of acrylic acid and styrene synthesized in the presence of BDB (a), PAADB (b), and PSDB (c). Each experiment was reproduced three times.

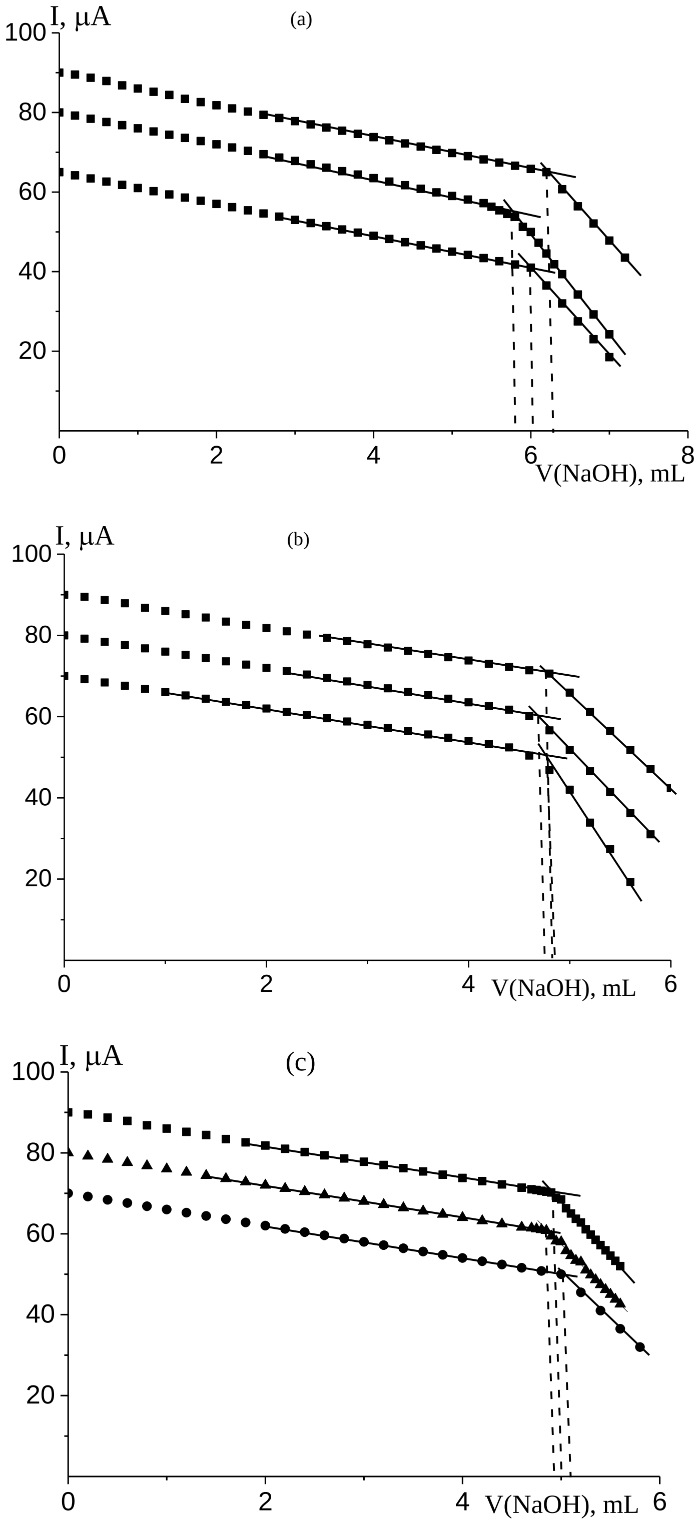

Figure. Curves of conductometric titration of the solutions of the copolymers of acrylic acid and styrene, synthesized in the presence of BDB (a), PAADB (b), and PSDB (c), in THF by 0.1 M of KOH.

The amount of KOH taken for titration is determined as intersection of the curves. The weight amount of acrylic acid is determined according to the equation:

$$w_{AA} = \frac{N_{KOH}V_{KOH}M_{AA} \cdot 100}{1000 \cdot m},$$

where N_{KOH} is normality of KOH, V_{KOH} is the volume of KOH taken for titration, M_{AA} is molar mass of acrylic acid, m is the weight of the sample of copolymer.

The results are summarized in the Table.

Table. The results of conductometric titration of the solutions of the RAFT-based copolymers of acrylic acid and styrene

**RAFT agent**	**m (g)**	**V_{NaOH}, mL**	**w_{AA}**
BDB	0.1	4.8	0.346
		4.7	0.339
		4.7	0.342
PAADB	0.15	6.2	0.298
		5.9	0.282
		6.0	0.290
PSDB	0.07	5.0	0.510
		4.9	0.505
		5.0	0.515

The procedure of estimation of the composition of the "grown" copolymer when PAA-based RAFT agents are used.

The weight fraction of AA in copolymer w_A can be defined as the sum of the weights of polymeric RAFT agent ($m_{polyRAFT}$) and weight of acrylic acid in "grown" copolymer (x) divided by the weight of the gross copolymer (m_G), i.e. the sum of the weights of polymeric RAFT agent and weight of "grown" copolymer (m_{gr}):

$$w_A = \frac{m_{polyRAFT} + x}{m_G} = \frac{m_{polyRAFT} + x}{m_{polyRAFT} + m_{gr}}$$

It allows to calculate x: $x = w_A(m_{polyRAFT} + m_{gr}) - m_{polyRAFT}$

Thus, the weight fraction of AA in "grown" copolymer ($w_{A,gr}$) may be determined as:

$$w_{A,gr} = \frac{x}{m_{gr}}$$

After that, the molar fraction of AA in grown copolymer may be evaluated.

The procedure of estimation of the composition of the "grown" copolymer when PS-based RAFT agents are used.

The weight fraction of styrene in copolymer w_S can be defined as the sum of the weights of polymeric RAFT agent ($m_{polyRAFT}$) and weight of styrene in "grown" copolymer (y) divided by the weight of the gross copolymer, i.e. the sum of the weights of polymeric RAFT agent and weight of "grown" copolymer (m_{gr}):

$$w_S = \frac{m_{polyRAFT} + y}{m_{polyRAFT} + m_{gr}}$$

It allows to calculate y: $y = w_S(m_{polyRAFT} + m_{gr}) - m_{polyRAFT}$

Thus, the weight fraction of styrene in "grown" copolymer ($w_{S,gr}$) may be determined as:

$$w_{S,gr} = \frac{y}{m_{gr}}$$

The weight fraction of acrylic acid in "grown" copolymer $w_{A,gr}$ is equal to $w_{A,gr} = 1 - w_{S,gr}$

After that, the molar fraction of AA in grown copolymer may be evaluated.

Fig. S1. The SEC curves normalized by unit area for copolymers formed from mixture of styrene (10 mol. %) and AA (90 mol. %) in the presence of BDB (a) and PAADB (b). AA : DMF = 1 : 1.5, $[AIBN]_0 = 1\times10^{-3}$, $[BDB]_0 = [PAADB]_0 = 6\times10^{-3}$ mol/L,*T* = 80ºC. (a) Conversions: 16.9 (*1*), 30.4 (*2*), 39.0 (*3*), 45.4 (*4*), and 50.2 % (*5*); (b) conversions: 12.2 (*1*), 18.8 (*2*), 26.6 (*3*), 33.9 (*4*), and 40.0 % (*5*).

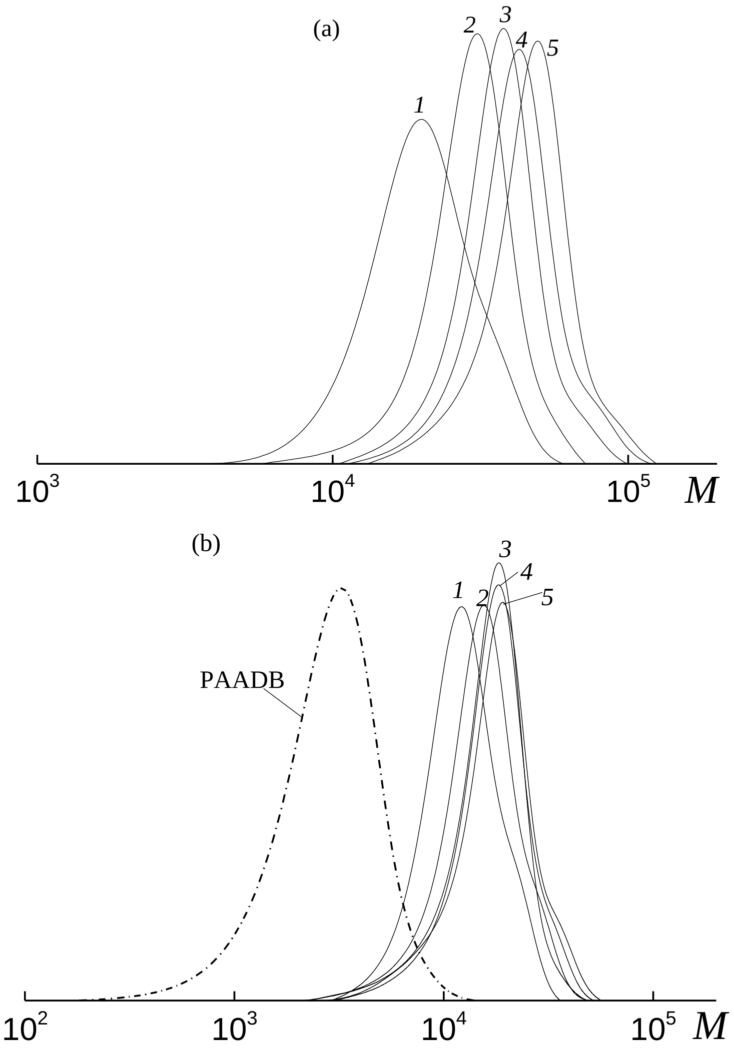

Fig. S2. The SEC curves normalized by unit area for copolymers formed from different monomer mixtures of styrene and AA in the presence of PSDB, AA : DMF = 1 : 3 mole, $[AIBN]_0 = 1\times10^{-3}$, $[PSDB]_0 = 6\times10^{-3}$ mol/L, *T* = 80°C. (a) f_{AA} = 0.15, conversions: 9.4 (*1*), 17.8 (*2*), 24.0 (*3*), 25.0 (*4*), and 30.4 % (*5*); (b) f_{AA} = 0.30, conversions: 8.8 (*1*), 12.0 (*2*), 17.0 (*3*), 20.6 (*4*), and 27.8 % (*5*); (c) f_{AA} = 0.85, conversions: 8.8 (*1*), 22.3 (*2*), 25.8 (*3*), 28.1 (*4*), and 32.0 % (*5*).

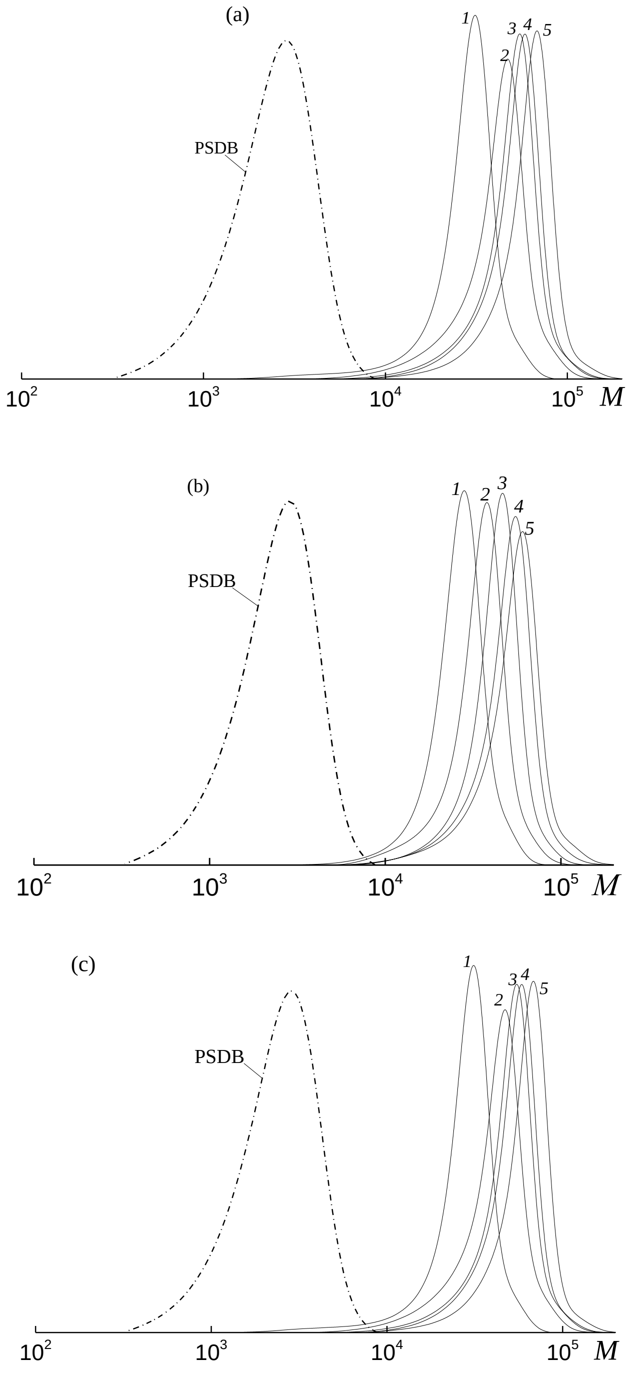

Fig. S3. Dependences of M_n (a) and M_w/M_n (b) from overall monomer conversion for copolymers formed from different monomer mixture of styrene and AA in the presence of BDB (*1*), PAADB (*2*), and PSDB (*3*–5), f_{AA} = 0.15 (*3*), 0.3 (*4*), 0.85 (*5*), 0.9 (*1, 2*), AA : DMF = 1 : 1.5 (*1, 2*) and 1 : 3 mole (*3*–5), $[AIBN]_0 = 1\times10^{-3}$, $[BDB]_0 = [PAADB]_0 = [PSDB]_0 = 6\times10^{-3}$ mol/L, *T* = 80°C.

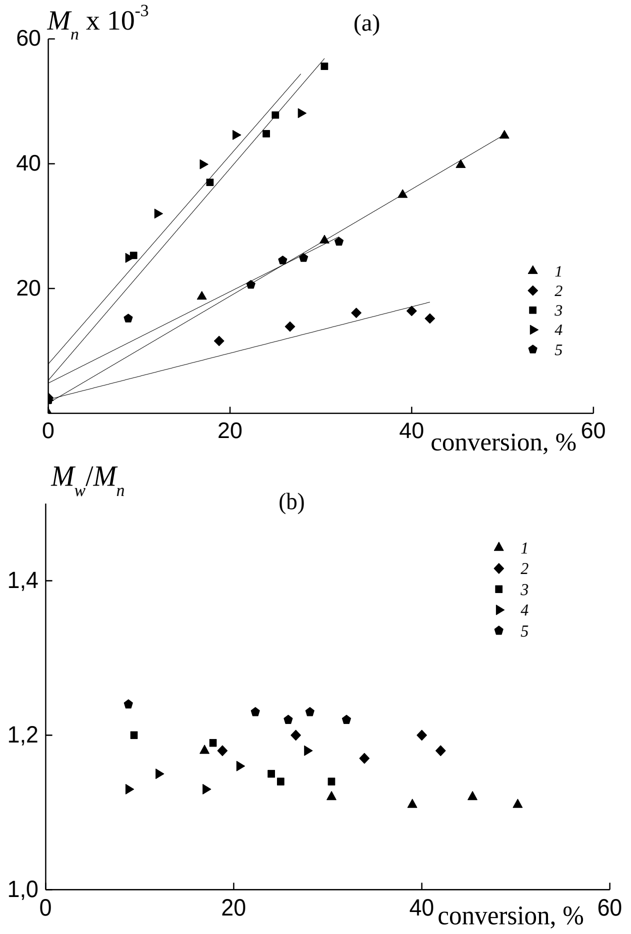

Fig. S4. Dependence of molar fraction of AA in grown copolymer (F_{AA}) from molar fraction AA in monomer feed (f_{AA}) for the copolymers formed in the presence PAADB1 (a), PAADB2 (b), and PAADB3 (c) at overall monomer conversion 2 (*1*), 5 (*2*), and 7 % (*3*); AA : DMF = 1 : 3, $[AIBN]_0 = 1\times10^{-3}$, $[PAADB1]_0 = [PAADB2]_0 = [PAADB3]_0 = 6\times10^{-3}$ mole/L, T = 80ºC.

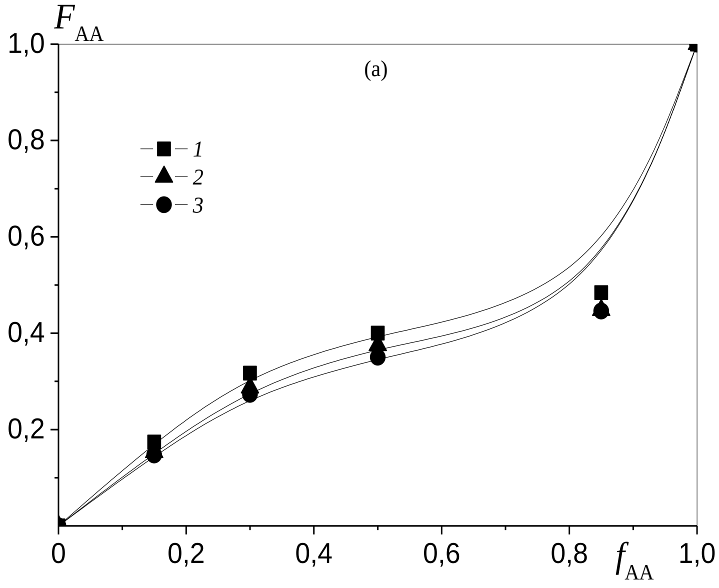

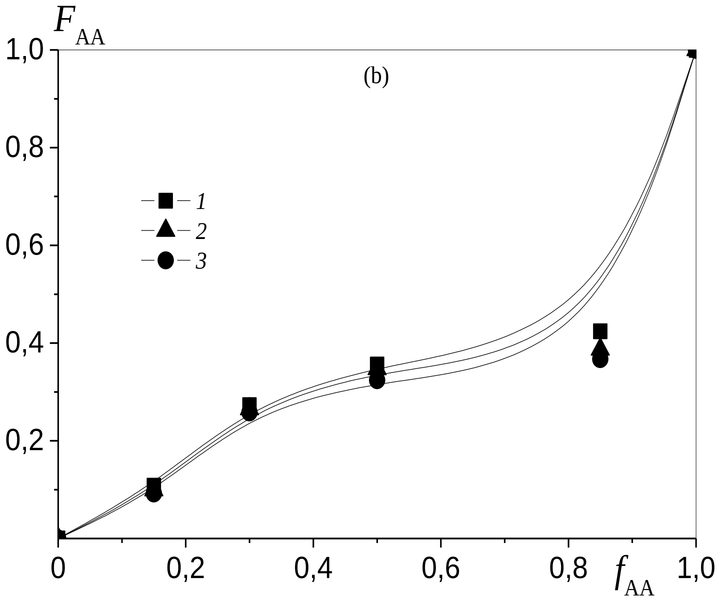

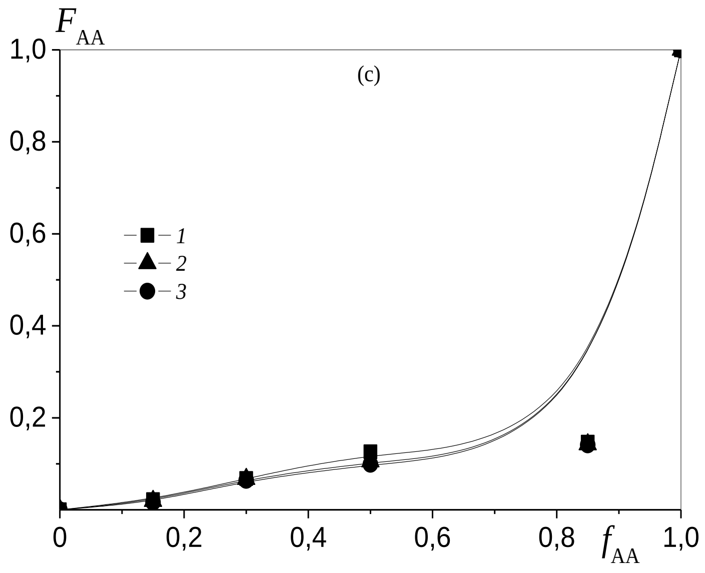
F AA
1,0
0,8
0,6
0,4
0,2
(c)
1
2
3
0
0,2
0,4
0,6
0,8
1,0
f AA

Fig. S5. The instantaneous diad composition $A_{AA}A_{AA}$ (*1, 3, 5*) and $A_{AA}A_S$ (*2, 4, 6*) calculated according to the terminal unit model for equimolar monomer mixture of AA and styrene using r_{AA} and r_S determined for the copolymerizations mediated by BDB (*1, 2*), PAADB (*3, 4*), and PSDB (*5, 6*).

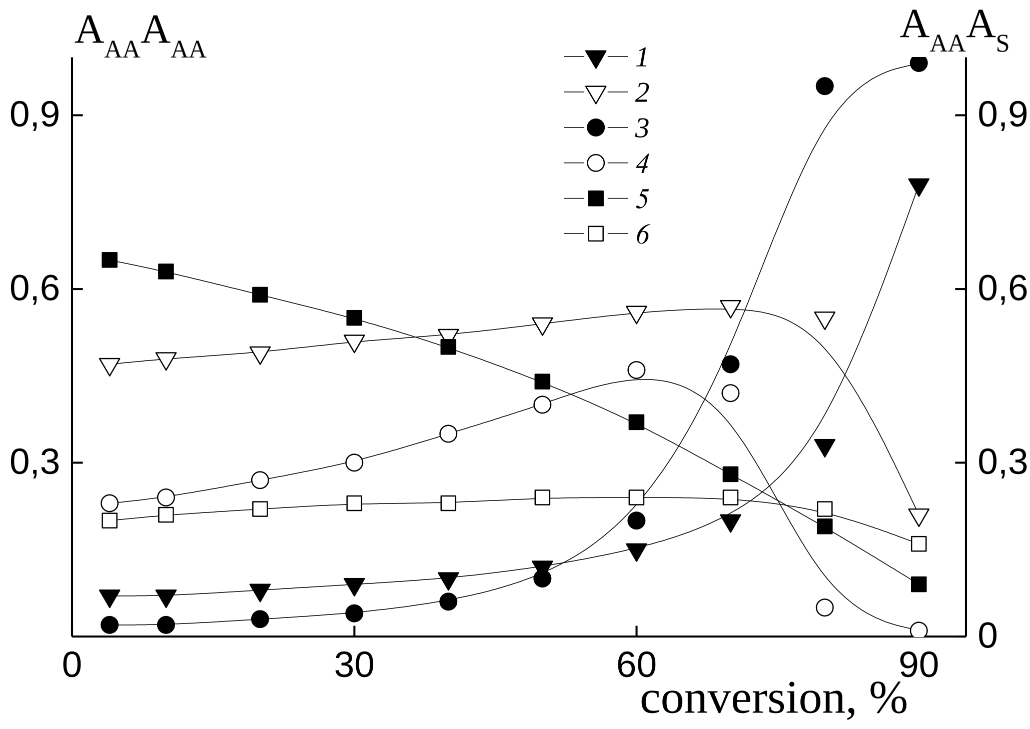

Fig. S6. The SEC curves normalized to the unit area for copolymers formed from mixture of styrene (10 mol. %) and AA (90 mol. %) in the presence of BTC (a), PAATC (b), and PSTC (c); AA : DMF = 1 : 1.57 mole (c), [AIBN]$_0$ = 1×10^{-3}, [BTC]$_0$ = [PAATC]$_0$ = [PSTC]$_0$ = 6×10^{-3} mole/L, *T* = 80°C. (a) Conversions: 8.0 (*1*), 12.6 (*2*), 18.7 (*3*), 30.0 (*4*), 47.0 (*5*), and 79.2 % (*6*); (b) conversions: 13.5 (*1*), 30.3 (*2*), 45.0 (*3*), and 65.0 % (*4*); (c) conversions: 11.4 (*1*), 12.3 (*2*), 28.0 (*3*), 47.0 (*4*), and 79.2 % (*5*).

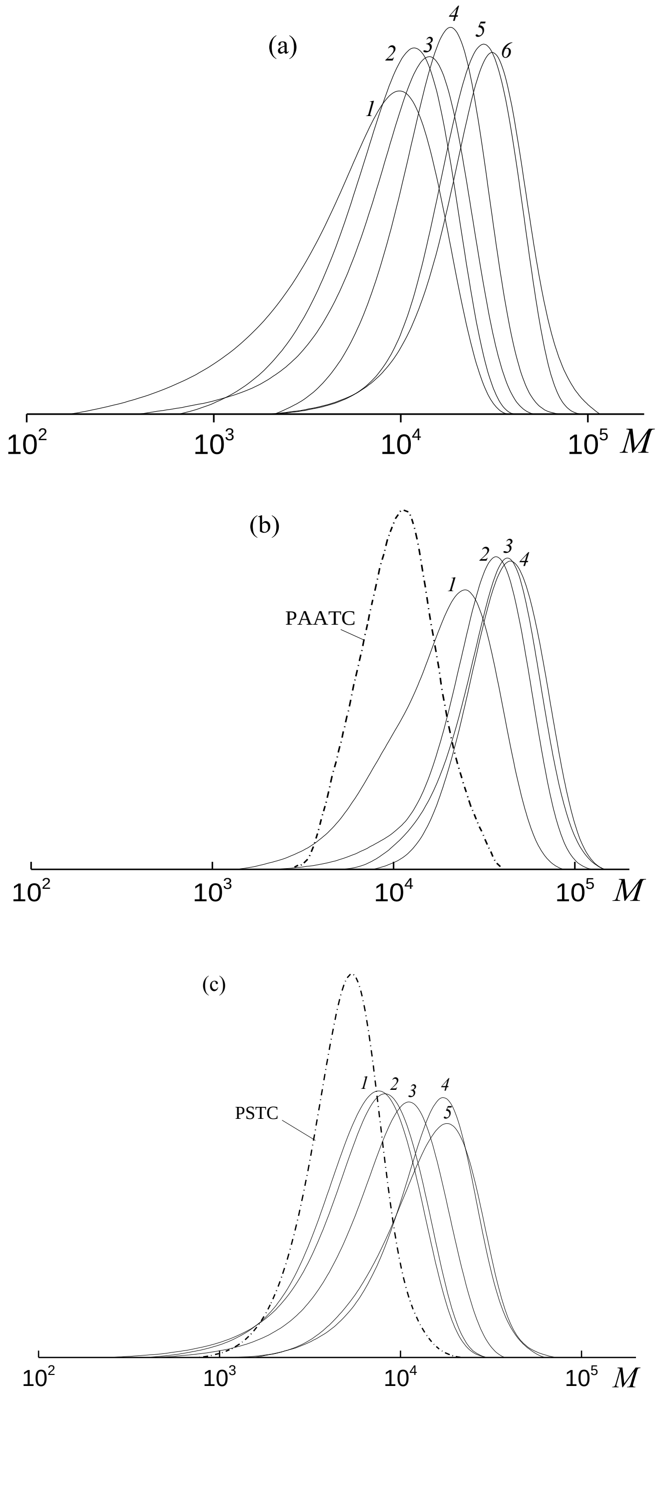

Fig. S7. Dependence of M_n (a) and M_w/M_n (b) from overall monomer conversion for copolymers formed from mixture of styrene (10 mol. %) and AA (90 mol. %) in the presence of BTC (*1*), PAATC (*2*), and PSTC (*3*); AA : DMF = 1 : 1.57; $[AIBN]_0 = 1\times10^{-3}$, $[BDB]_0 = [PAADB]_0 = [PSDB]_0 = 6\times10^{-3}$ mole/L, $T = 80^oC$.

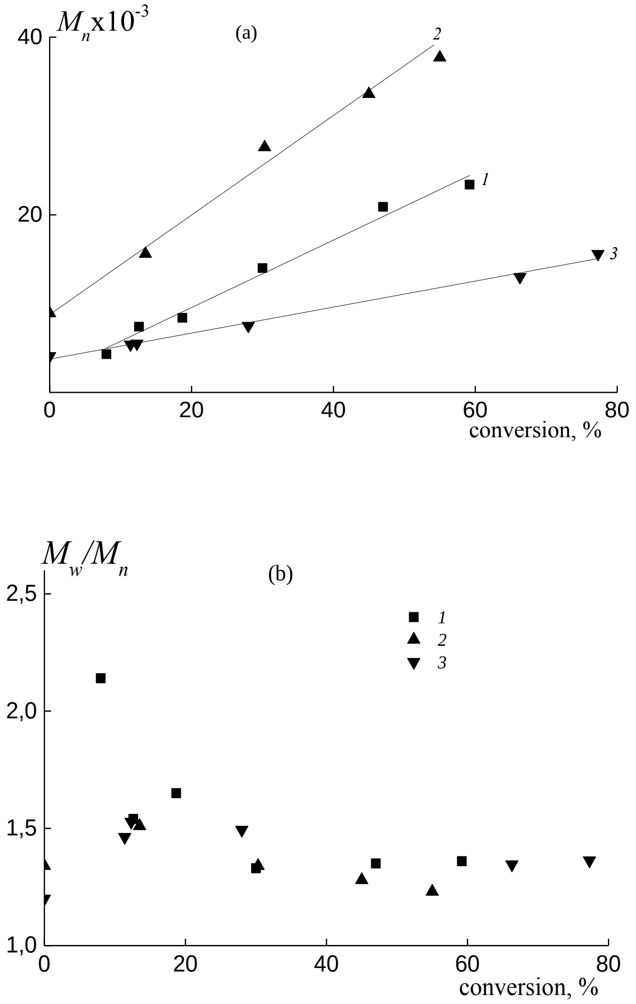

Fig. S8. The SEC curves normalized to the unit area for copolymers formed from mixture of styrene (10 mol. %) and AA (90 mol. %) in the presence of BTC (*1*), PAATC (*3*), and PSTC (*5*) and the same copolymers after heating with the 100-fold excess of AIBN (*2*, *4*, *6*).

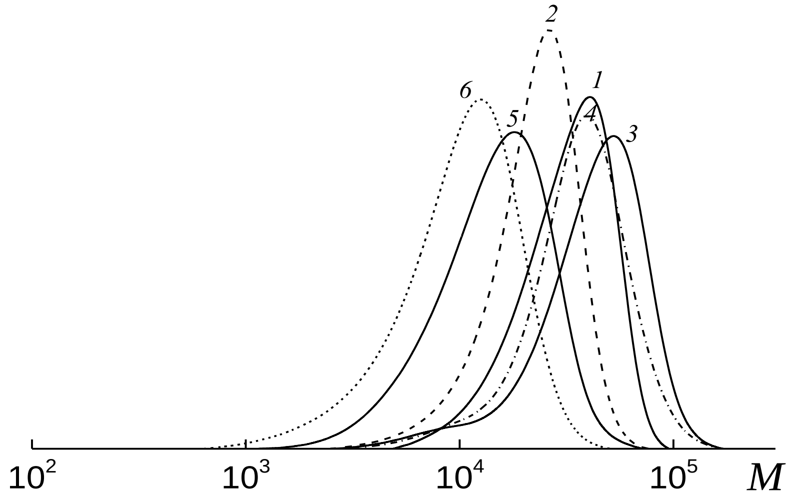

Supplement: RA-008-C8RA00048D-s001 [file RA-008-C8RA00048D-s001.pdf]
